# Supplementary material for: The effects of weather and mobility on respiratory viruses dynamics before and during the COVID-19 pandemic in the USA and Canada
Source: PLOS Digit Health. 2023 Dec 21;2(12):e0000405. doi: 10.1371/journal.pdig.0000405 (PMC10734953; doi:10.1371/journal.pdig.0000405)
Supplement: S1 Fig — (PDF) [file pdig.0000405.s001.pdf]

A.

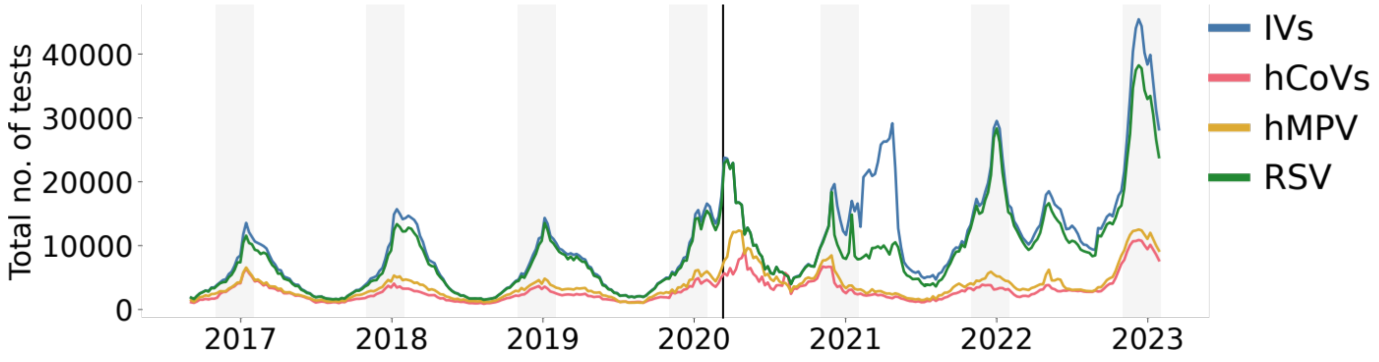

B.

Virus	Correlation coefficient
IVA	0.809012
IVB	0.917524
hCoVs	0.802461
hMPV	0.785859
RSV	0.743064

S1 Fig. (A) Total number of tests performed in Canada for all viruses from September 2016 until January 2023. Note the number of test for IVs is not reported individually but for both IVA and IVB together. Shaded areas correspond to the period between November and February; solid vertical line marks the WHO pandemic declaration, in March 11th, 2020. **(B)** Pearson correlation coefficient for each virus incidence (calculated with the positivity rate and ILI outpatient rates) and the incidence proxy calculated from the number of positive test and the average Canadian population between 2016 and 2023.
