# Supplementary material for: The effects of weather and mobility on respiratory viruses dynamics before and during the COVID-19 pandemic in the USA and Canada
Source: PLOS Digit Health. 2023 Dec 21;2(12):e0000405. doi: 10.1371/journal.pdig.0000405 (PMC10734953; doi:10.1371/journal.pdig.0000405)
Supplement: S2 Fig — (PDF) [file pdig.0000405.s002.pdf]

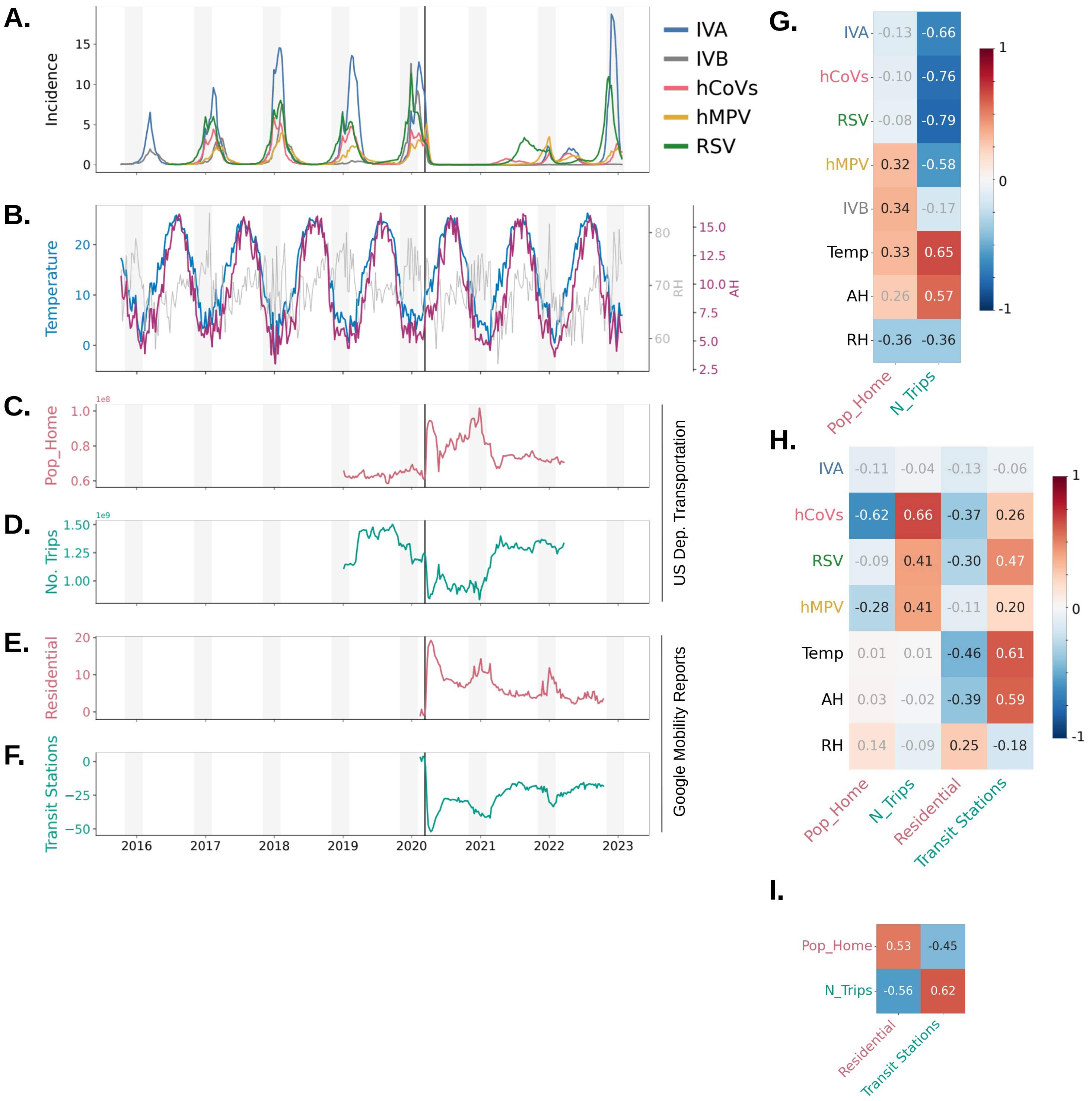

S2 Fig. Relevant time series (**A** to **F**) and corresponding correlations (**G** to **I**) for the USA, between 2015 and 2023. **(A)** Incidence for all viruses; **(B)** temperature, AH and RH; **(C)** population at home; **(D)** number of trips; **(E)** residential time; **(F)** transit station visitors. Please note that mobility datasets start later (see Methods). Shaded areas correspond to the periods between November and February; solid vertical line marks the WHO pandemic declaration, in March 11th, 2020. Pearson correlation coefficients between **(G)** incidence of the different viruses, weather and mobility variables from 2019 to 2020 (pre-COVID19); **(H)** incidence of the different viruses, weather and mobility variables from 2020 to 2022 (pandemic period); **(I)** different mobility measures, between 2020 and 2022 (pandemic period). Coefficients in white or black, p-value≤0.05; coefficients in light grey, non-significant
