# Supplementary material for: The effects of weather and mobility on respiratory viruses dynamics before and during the COVID-19 pandemic in the USA and Canada
Source: PLOS Digit Health. 2023 Dec 21;2(12):e0000405. doi: 10.1371/journal.pdig.0000405 (PMC10734953; doi:10.1371/journal.pdig.0000405)
Supplement: S3 Fig — (PDF) [file pdig.0000405.s003.pdf]

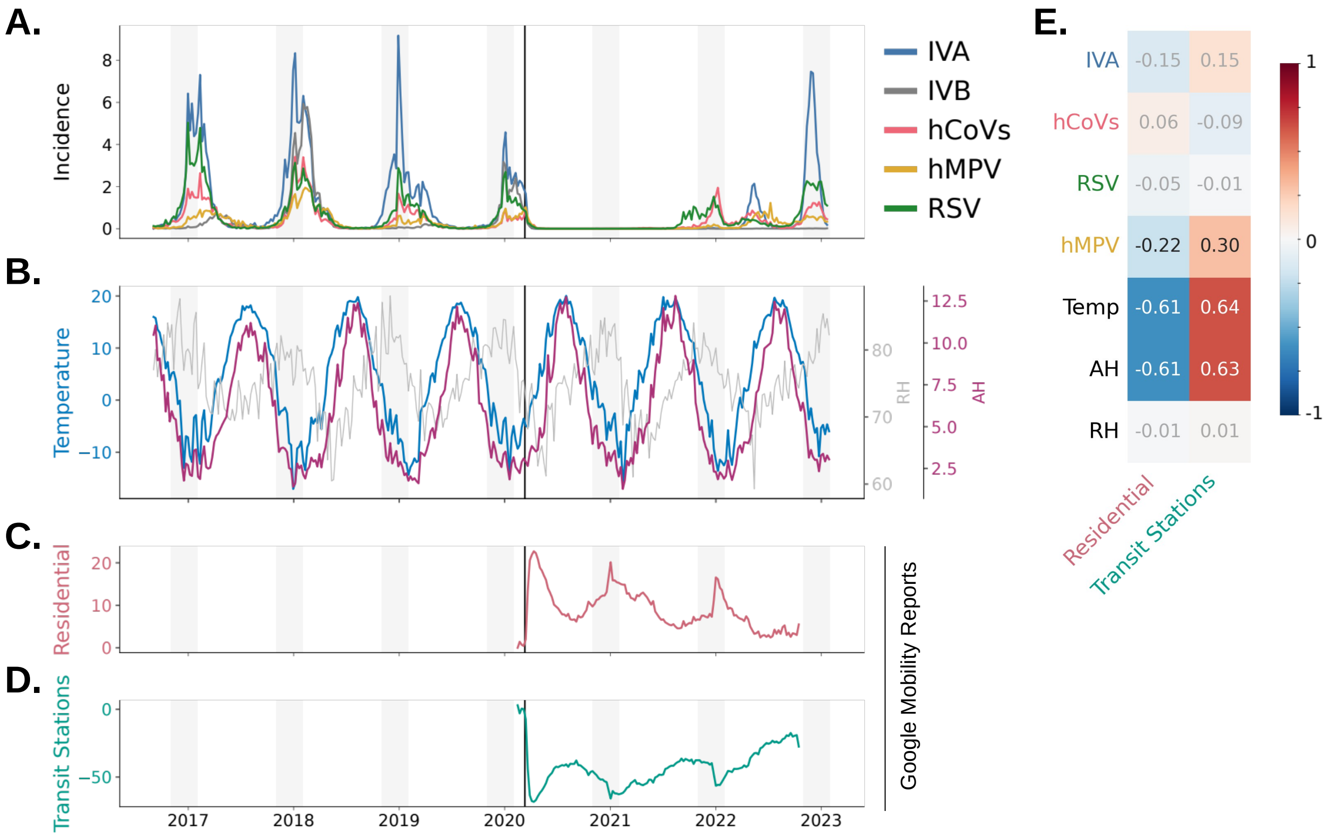

S3 Fig. Relevant time series (**A** to **D**) and corresponding correlations (**E**) for Canada, **(A)** Incidence for all viruses between 2016 and 2013; **(B)** temperature, AH and RH between 2016 and 2013; **(C)** **r**esidential time; **(D)** transit station visitors between 2020 and 2022. Shaded areas correspond to the periods between November and February, solid vertical line marks the WHO pandemic declaration, in March 11th, 2020. **(E)** Pearson correlation coefficients between the incidence of the different viruses, weather and different mobility measures between 2020 and 2022 (pandemic period). Coefficients in white or black, p-value≤0.05; coefficients in light grey, non-significant
