# Supplementary material for: The effects of weather and mobility on respiratory viruses dynamics before and during the COVID-19 pandemic in the USA and Canada
Source: PLOS Digit Health. 2023 Dec 21;2(12):e0000405. doi: 10.1371/journal.pdig.0000405 (PMC10734953; doi:10.1371/journal.pdig.0000405)
Supplement: S4 Fig — PCA analysis for the US Department of Transportation data set for mobility variable selection. (PDF) [file pdig.0000405.s004.pdf]

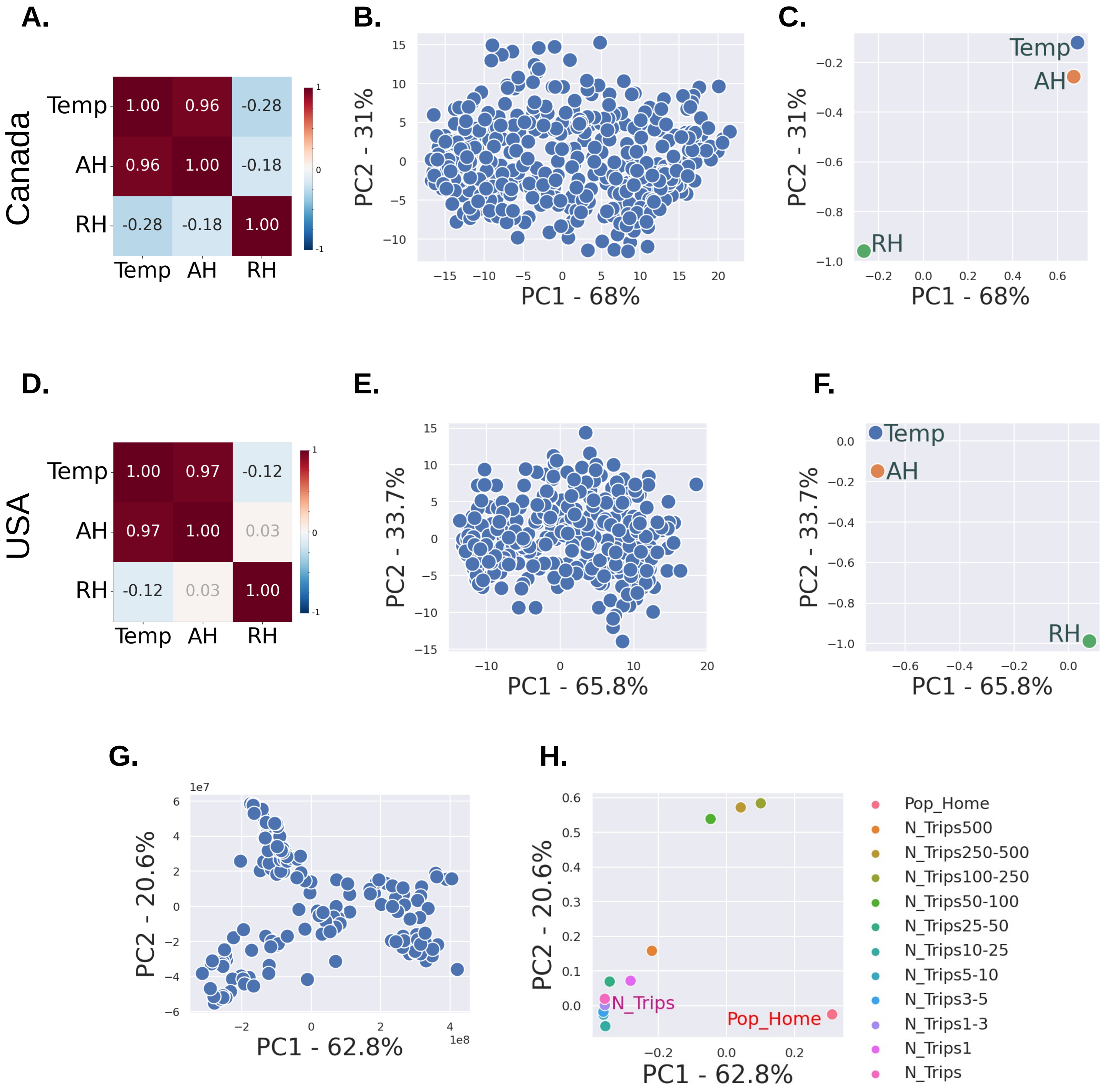

S4 Fig. **(A)** and **(D)** Pearson correlation coefficients for the weather time series in Canada and the USA, respectively, for the whole study period. Correlation coefficients in white or black, p-value≤0.05; coefficients in light grey, non-significant. **(B)** and **(E)** principal component 1 (PC1) and principal component 2 (PC2) for the weather variables in Canada and the USA, respectively. The percentage of variation explained by each PC is shown in the axis. **(C)** and **(F)** loading plots showing the correlation coefficients between each weather variable and PC1 and PC2, (i.e., the elements of the eigenvector) for Canada and the USA, respectively. **(G)** PC1 and PC2 for the US Department of Transportation mobility variables. The percentage of variation explained by each PC is shown in the axis. **(H)** loading plot showing the correlation coefficients between each mobility variable in the dataset and PC1 and PC2, (i.e., the elements of the eigenvector).
