# Supplementary material for: The effects of weather and mobility on respiratory viruses dynamics before and during the COVID-19 pandemic in the USA and Canada
Source: PLOS Digit Health. 2023 Dec 21;2(12):e0000405. doi: 10.1371/journal.pdig.0000405 (PMC10734953; doi:10.1371/journal.pdig.0000405)
Supplement: S5 Fig — (PDF) [file pdig.0000405.s005.pdf]

A.

Canada

Virus	Model	ΔAIC
IVA	0	0.0
	1	73.0
	2	21.0
	3	-22.0
	4	-43.0
RSV	0	0.0
	1	72.0
	2	30.0
	3	12.0
	4	-5.0
hCoVs	0	0.0
	1	99.0
	2	68.0
	3	34.0
	4	10.0
IVB	0	0.0
	1	107.0
	2	74.0
	3	32.0
	4	-3.0
hMPV	0	0.0
	1	119.0
	2	87.0
	3	54.0
	4	25.0

B.

USA

Virus	Model	ΔAIC
IVA	0	0.0
	1	229.0
	2	169.0
	3	100.0
	4	44.0
RSV	0	0.0
	1	101.0
	2	41.0
	3	-3.0
	4	-26.0
hCoVs	0	0.0
	1	140.0
	2	100.0
	3	58.0
	4	31.0
IVB	0	0.0
	1	192.0
	2	105.0
	3	66.0
	4	27.0
hMPV	0	0.0
	1	189.0
	2	147.0
	3	103.0
	4	69.0

C.

Virus	Model	ΔAIC
IVA	0	0.0
	1	73.0
	2	58.0
	3	44.0
	4	31.0
RSV	0	0.0
	1	132.0
	2	116.0
	3	101.0
	4	89.0
hCoVs	0	0.0
	1	108.0
	2	82.0
	3	59.0
	4	44.0
hMPV	0	0.0
	1	115.0
	2	97.0
	3	93.0
	4	74.0

D.

Virus	Model	ΔAIC
IVA	0	0.0
	1	99.0
	2	77.0
	3	51.0
	4	32.0
RSV	0	0.0
	1	124.0
	2	110.0
	3	92.0
	4	75.0
hCoVs	0	0.0
	1	146.0
	2	99.0
	3	72.0
	4	52.0
hMPV	0	0.0
	1	105.0
	2	78.0
	3	57.0
	4	41.0

S5 Fig. ΔAIC for the temperature and RH models that include: (0) no auto-correlation variable (AC), (1) 1-week lag AC, (2) 2-week lag AC (3) 3-week lag AC, and (4) 4-week lag AC for **(A)** Canada pre-COVID-19 period, **(B)** USA pre-COVID-19 period, **(C)** Canada pandemic period, **(D)** USA pandemic period.
