# Supplementary material for: The effects of weather and mobility on respiratory viruses dynamics before and during the COVID-19 pandemic in the USA and Canada
Source: PLOS Digit Health. 2023 Dec 21;2(12):e0000405. doi: 10.1371/journal.pdig.0000405 (PMC10734953; doi:10.1371/journal.pdig.0000405)
Supplement: S6 Fig — (PDF) [file pdig.0000405.s006.pdf]

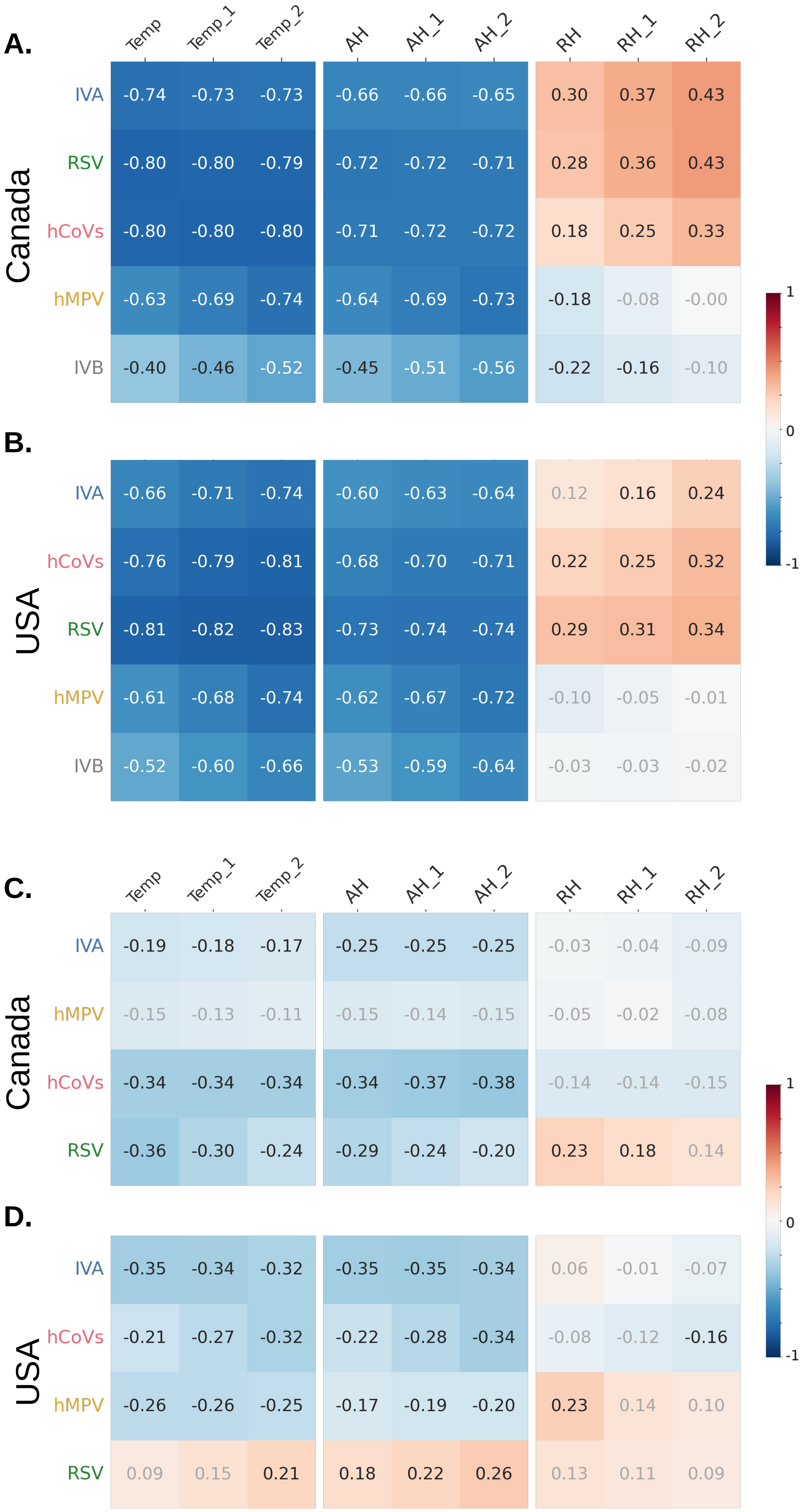

S6 Fig. Pearson correlation coefficients between all the viruses and the weather variables lagged 0 (no lag), 1 (marked as _1) or 2 (marked as _2) weeks for **(A)** Canada pre-COVID-19 period, **(B)** USA pre-COVID-19 period, **(C)** Canada pandemic period, **(D)** USA pandemic period. Coefficients in white or black, p-value≤0.05; coefficients in light grey, non-significant
