# Supplementary material for: The effects of weather and mobility on respiratory viruses dynamics before and during the COVID-19 pandemic in the USA and Canada
Source: PLOS Digit Health. 2023 Dec 21;2(12):e0000405. doi: 10.1371/journal.pdig.0000405 (PMC10734953; doi:10.1371/journal.pdig.0000405)
Supplement: S8 Fig — (PDF) [file pdig.0000405.s008.pdf]

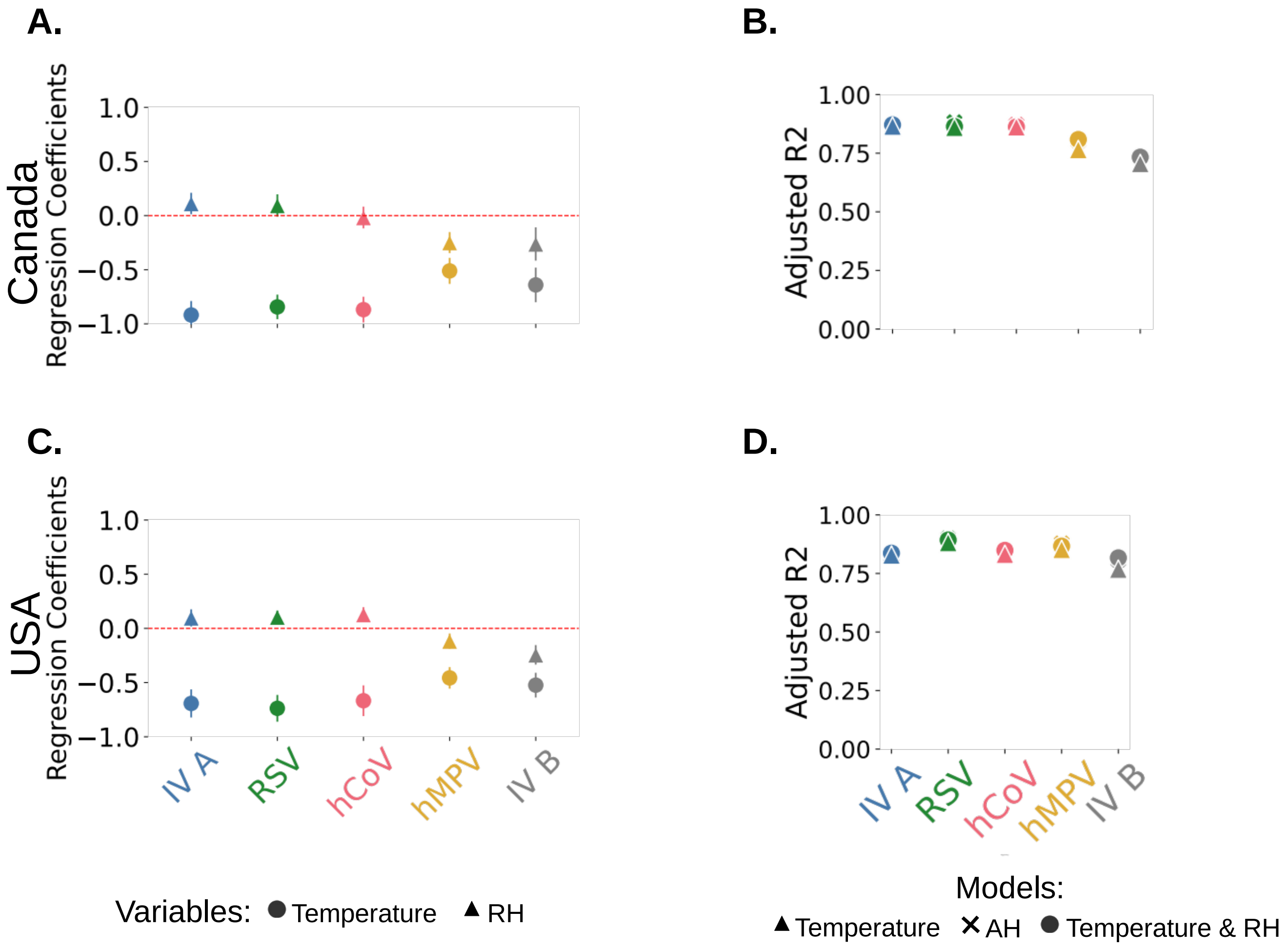

S8 Fig. **(A)** and **(C)** regression coefficients for the temperature and RH model with 95% confidence intervals in the pre-COVID-19 pandemic period from September 2016 to September 2018 in Canada and the USA, respectively. Coefficients for the AC term are not represented. **(B)** and **(D)** pseudo-R^2 for the best model (square), temperature (triangle), AH (cross) and temperature-RH (circle) models for the pre-COVID-19 pandemic period from September 2016 to September 2018 in Canada and the USA, respectively.
