# Supplementary material for: The effects of weather and mobility on respiratory viruses dynamics before and during the COVID-19 pandemic in the USA and Canada
Source: PLOS Digit Health. 2023 Dec 21;2(12):e0000405. doi: 10.1371/journal.pdig.0000405 (PMC10734953; doi:10.1371/journal.pdig.0000405)
Supplement: S9 Fig — (PDF) [file pdig.0000405.s009.pdf]

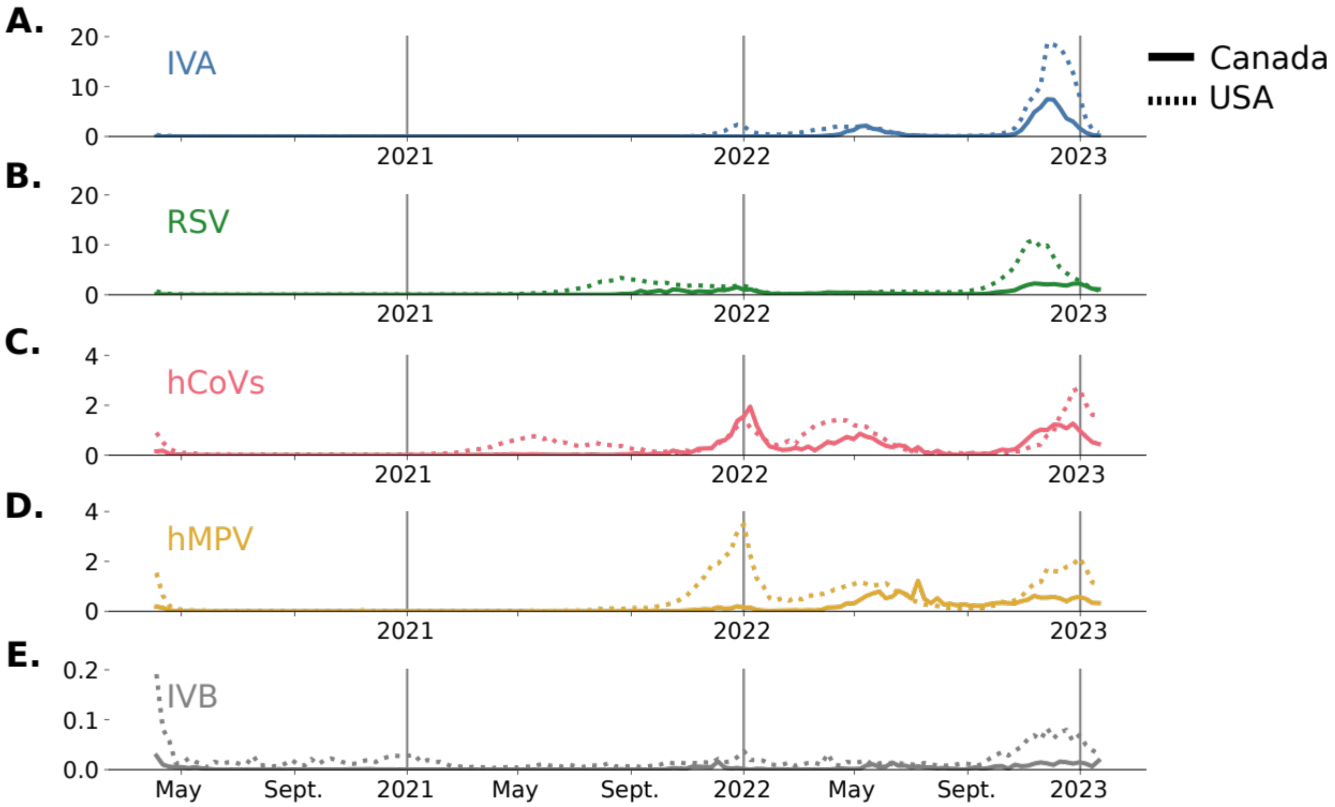

S9 Fig. Incidence of (**A**) IVA, (**B**) RSV, (**C**) hCoVs, (**D**) hMPV, and (**E**) IVB, in Canada and the USA, in the pandemic period (starting at April 1st, 2020). Solid lines, incidence for Canada; dotted lines, incidence for the USA. Y axes have different scales for the different viruses. Solid grey vertical lines mark 1st of January.
