# Supplementary material for: The effects of weather and mobility on respiratory viruses dynamics before and during the COVID-19 pandemic in the USA and Canada
Source: PLOS Digit Health. 2023 Dec 21;2(12):e0000405. doi: 10.1371/journal.pdig.0000405 (PMC10734953; doi:10.1371/journal.pdig.0000405)
Supplement: S10 Fig — (PDF) [file pdig.0000405.s010.pdf]

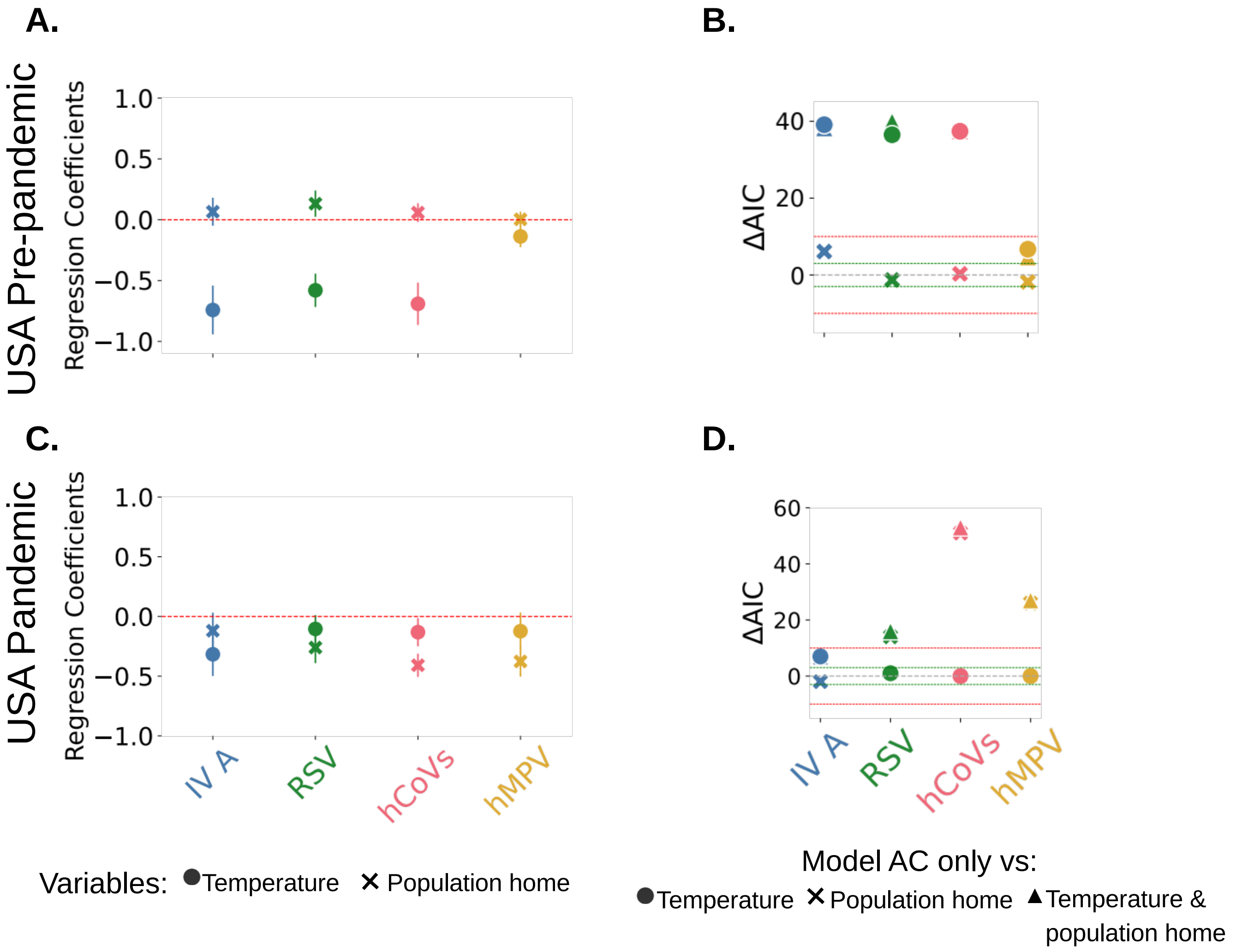

S10 Fig. **(A)** and **(C)** regression coefficients with 95% confidence intervals for the temperature (circle) and population at home (cross) model with 95% confidence intervals for the USA pre-COVID-19 pandemic and pandemic periods, respectively. **(B)** and **(D)** AIC difference (ΔAIC) between the AC only model and the temperature only model (circle), the population at home only model (cross) and the temperature and population at home model (triangle) for the pre-COVID-19 pandemic and pandemic periods, respectively.
