# Supplementary material for: The effects of weather and mobility on respiratory viruses dynamics before and during the COVID-19 pandemic in the USA and Canada
Source: PLOS Digit Health. 2023 Dec 21;2(12):e0000405. doi: 10.1371/journal.pdig.0000405 (PMC10734953; doi:10.1371/journal.pdig.0000405)
Supplement: S11 Fig — (PDF) [file pdig.0000405.s011.pdf]

A.

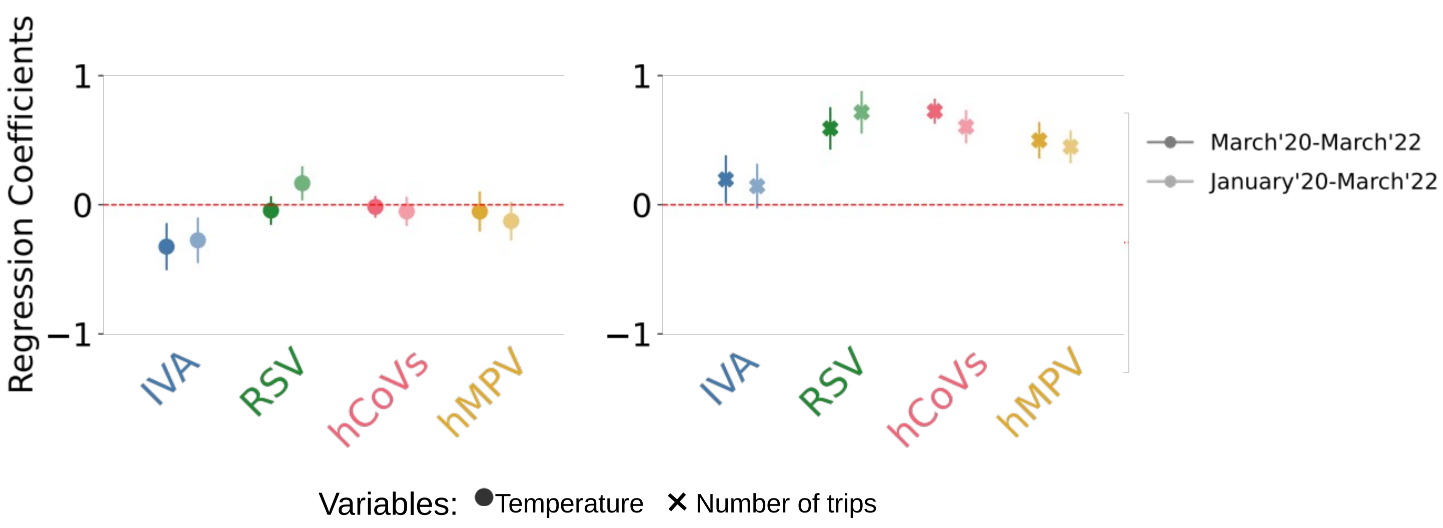

B.

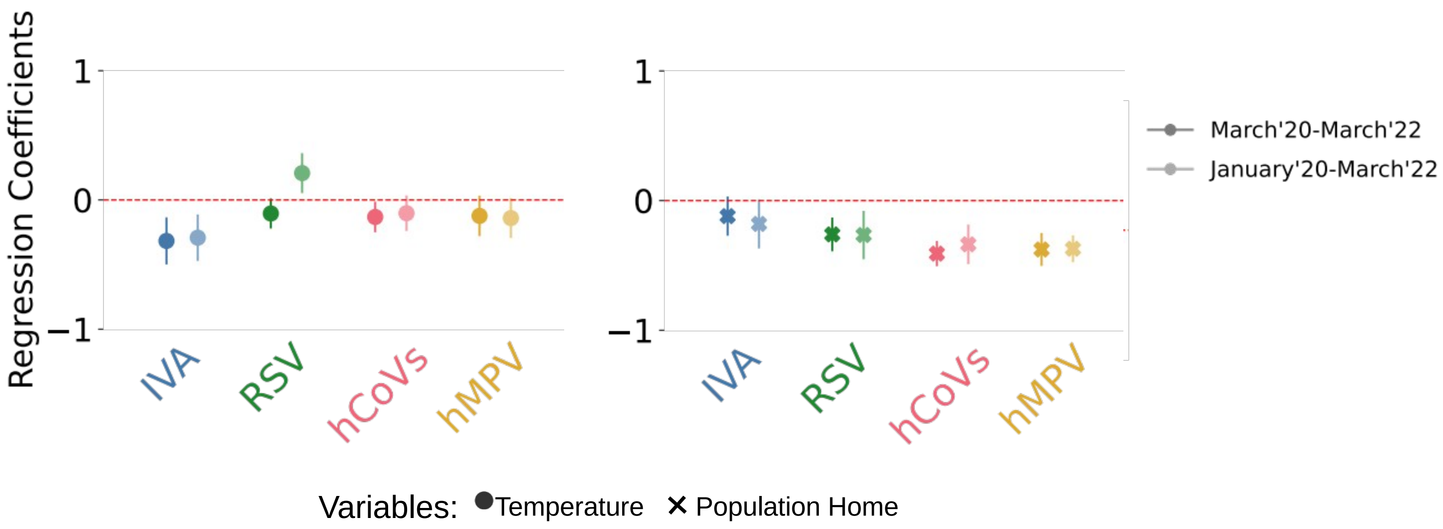

S11 Fig. Comparison of the regression coefficients with 95% confidence intervals for **(A)** temperature (circle) and number of trips (cross) model or **(B)** temperature (circle) and population at home (cross) model, when the pandemic period starts in January 2020 (faint colors) or March 2020 (vivid colors) for the USA.
