# Supplementary material for: The effects of weather and mobility on respiratory viruses dynamics before and during the COVID-19 pandemic in the USA and Canada
Source: PLOS Digit Health. 2023 Dec 21;2(12):e0000405. doi: 10.1371/journal.pdig.0000405 (PMC10734953; doi:10.1371/journal.pdig.0000405)
Supplement: S12 Fig — Regression coefficients for the temperature and number of trips model with dummy pandemic variable and interactions. (PDF) [file pdig.0000405.s012.pdf]

A.

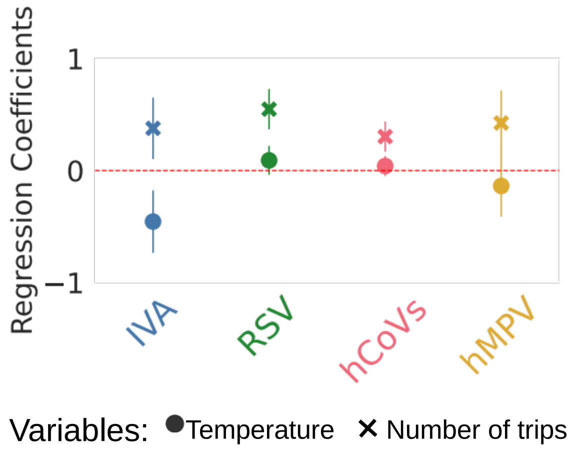

B.

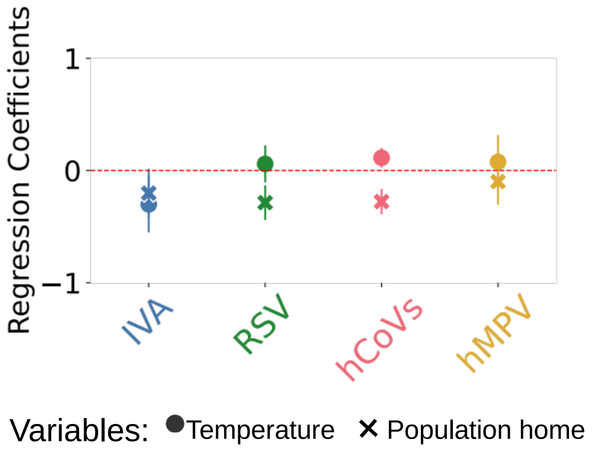

C.

Virus	Pandemic	Temperature	Temperature : Pandemic	Trips	Trips : Pandemic
**IVA**	-2.1 **	-0.53 **	0.3 ns	-0.93 **	1.05 **
**RSV**	-0.99 **	-0.8 **	1.08 **	-0.29 ns	1.1 **
**hCoVs**	-1.16 **	-0.62 **	0.51 **	-0.9 **	1.55 **
**IVB**	-1.03 **	-0.64 **	0.59 **	0.26 ns	-0.35 ns
**hMPV**	-1.43 **	-0.24 ns	0.03 ns	-0.29 ns	0.68 *

S12 Fig. Regression coefficients with 95% confidence intervals for the **(A)** temperature (circle) and number of trips (cross) model and **(B)** temperature (circle) and population at home (cross) model, with a shorter pandemic period (15 months, January 2021-March 2022). **(C)** Regression coefficients for the temperature and number of trips model with dummy pandemic variable and interactions terms (depicted with ":"). **, p-value≤0.01; *, p-value≤0.05; ns, non-significant.
