# Supplementary material for: The effects of weather and mobility on respiratory viruses dynamics before and during the COVID-19 pandemic in the USA and Canada
Source: PLOS Digit Health. 2023 Dec 21;2(12):e0000405. doi: 10.1371/journal.pdig.0000405 (PMC10734953; doi:10.1371/journal.pdig.0000405)
Supplement: S13 Fig — (PDF) [file pdig.0000405.s013.pdf]

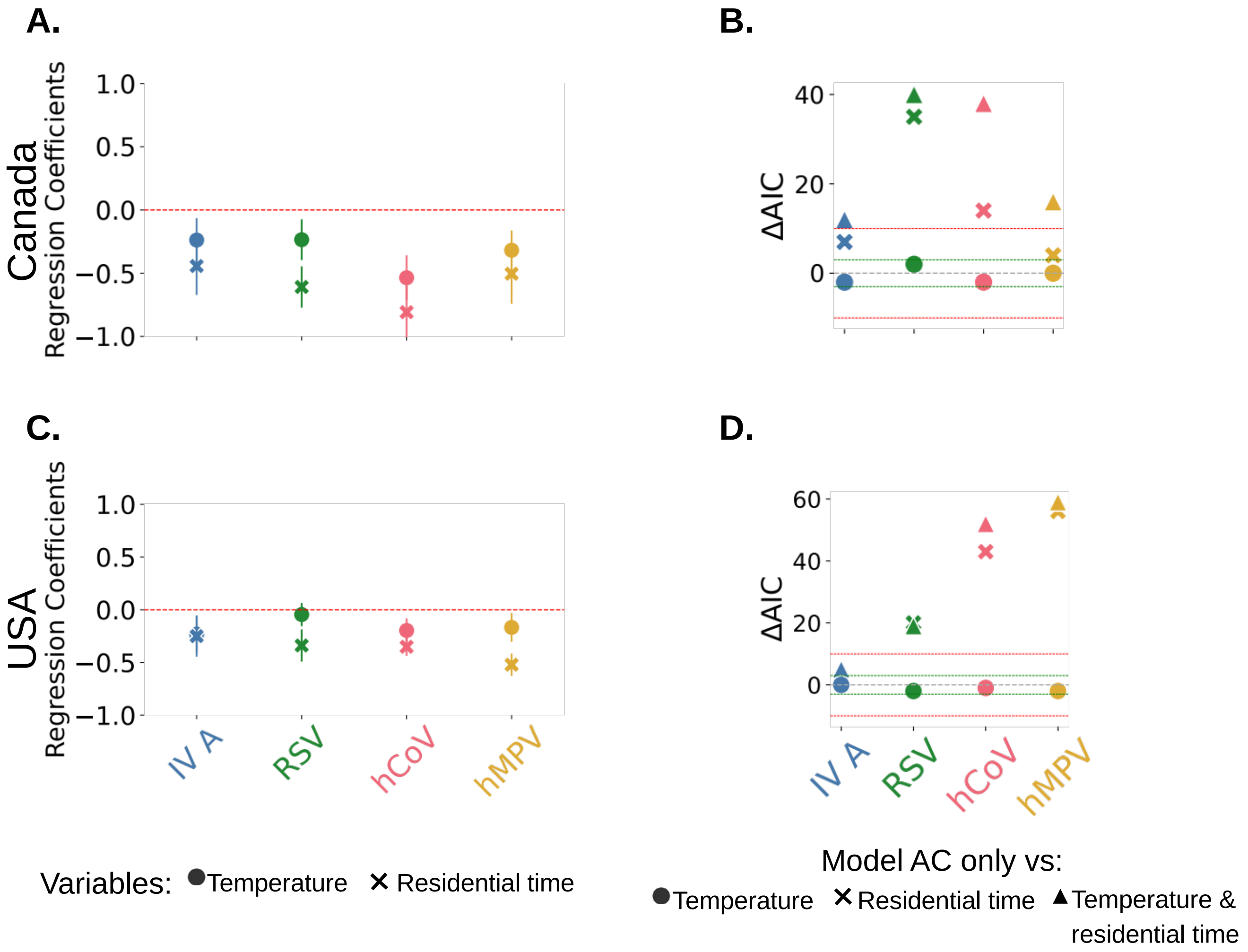

S13 Fig. (A) and **(C)** regression coefficients for the temperature (circle) and residential time (cross) models with 95% confidence intervals for the pandemic period in Canada and the USA, respectively. **(B)** and **(D)** AIC difference (ΔAIC) between the auto-correlation (AC) only model and the temperature only model (circle), the residential time only model (cross) and the temperature-residential time model (triangle) for the pandemic period in Canada and the USA, respectively.
