# Supplementary material for: The effects of weather and mobility on respiratory viruses dynamics before and during the COVID-19 pandemic in the USA and Canada
Source: PLOS Digit Health. 2023 Dec 21;2(12):e0000405. doi: 10.1371/journal.pdig.0000405 (PMC10734953; doi:10.1371/journal.pdig.0000405)
Supplement: S1 Table — (PDF) [file pdig.0000405.s014.pdf]

S1 Table. Regression models results for the weather analysis for Canada and the USA pre-COVID-19 pandemic (2015/16-2020).

pValueAC, p-value autocorrelation(AC) Breusch-Godfrey test up to 53 weeks; Coef, regression coefficient; Temp, temperature; AH, absolute humidity; RH, relative humidity; Yt-1, AC term 1 week; Yt-1/2 AC term 1 and 2 weeks. Models used for comparison in grey.

CANADA (2015/16-2020)

Virus	Model	AIC	R²	pValueAC	Variable1	Coef1	pValue1	error1	Variable2	Coef2	pValue2	error2	Variable3	Coef3	pValue3	error3	Variable4	Coef4	pValue4	error4	Variable5	Coef5	pValue5	error5	Variable6	Coef6	pValue6	error6
IVA	None	-2096.0	0.0	0.0	Intercept	-6.55	0.0	0.2	Precision	5.94	0.0	0.26																
IVA	Yt-1	-2243.0	0.59	0.34	Intercept	-6.86	0.0	0.13	Yt-1	0.67	0.0	0.06	Precision	6.96	0.0	0.23												
IVA	Temp	-2309.0	0.69	0.0	Intercept	-7.0	0.0	0.13	Temp	-0.99	0.0	0.1	Precision	7.35	0.0	0.23												
IVA	AH	-2284.0	0.64	0.0	Intercept	-7.0	0.0	0.14	AH	-1.09	0.0	0.14	Precision	7.26	0.0	0.24												
IVA	Yt-1/2	-2239.0	0.62	0.0	Intercept	-6.89	0.0	0.13	Yt-1	0.51	0.0	0.11	Yt-2	0.22	0.0	0.12	Precision	7.06	0.0	0.23								
IVA	Temp_Yt-1	-2382.0	0.81	0.19	Intercept	-7.13	0.0	0.1	Temp	-0.77	0.0	0.1	Yt-1	0.37	0.0	0.06	Precision	7.9	0.0	0.22								
IVA	AH_Yt-1	-2397.0	0.83	0.04	Intercept	-7.21	0.0	0.11	AH	-0.93	0.0	0.13	Yt-1	0.42	0.0	0.05	Precision	8.08	0.0	0.22								
IVA	Temp_RH	-2309.0	0.69	0.0	Intercept	-7.01	0.0	0.13	Temp	-0.99	0.0	0.1	RH	0.06	0.24	0.1	Precision	7.37	0.0	0.23								
IVA	AH_RH	-2288.0	0.66	0.0	Intercept	-7.02	0.0	0.14	AH	-1.09	0.0	0.14	RH	0.11	0.01	0.09	Precision	7.32	0.0	0.24								
IVA	Temp_Yt-1/2	-2367.0	0.82	0.0	Intercept	-7.13	0.0	0.11	Temp	-0.75	0.0	0.1	Yt-1	0.33	0.0	0.08	Yt-2	0.08	0.09	0.09	Precision	7.92	0.0	0.22				
IVA	AH_Yt-1/2	-2382.0	0.83	0.0	Intercept	-7.21	0.0	0.11	AH	-0.9	0.0	0.13	Yt-1	0.37	0.0	0.07	Yt-2	0.07	0.07	0.08	Precision	8.1	0.0	0.22				
IVA	Temp_RH_Yt-1	-2382.0	0.81	0.21	Intercept	-7.14	0.0	0.11	Temp	-0.77	0.0	0.1	RH	0.06	0.17	0.09	Yt-1	0.37	0.0	0.06	Precision	7.92	0.0	0.22				
IVA	AH_RH_Yt-1	-2400.0	0.83	0.13	Intercept	-7.23	0.0	0.11	AH	-0.94	0.0	0.13	RH	0.09	0.02	0.07	Yt-1	0.4	0.0	0.05	Precision	8.13	0.0	0.23				
IVA	Temp_RH_Yt-1/2	-2367.0	0.82	0.0	Intercept	-7.14	0.0	0.11	Temp	-0.75	0.0	0.1	RH	0.07	0.12	0.09	Yt-1	0.32	0.0	0.08	Yt-2	0.08	0.06	0.09	Precision	7.94	0.0	0.22
IVA	AH_RH_Yt-1/2	-2386.0	0.84	0.0	Intercept	-7.23	0.0	0.11	AH	-0.92	0.0	0.13	RH	0.1	0.01	0.07	Yt-1	0.35	0.0	0.07	Yt-2	0.08	0.04	0.08	Precision	8.15	0.0	0.23
RSV	None	-2311.0	0.0	0.0	Intercept	-7.18	0.0	0.19	Precision	6.65	0.0	0.25																
RSV	Yt-1	-2454.0	0.58	0.06	Intercept	-7.45	0.0	0.13	Yt-1	0.61	0.0	0.05	Precision	7.6	0.0	0.22												
RSV	Temp	-2539.0	0.71	0.0	Intercept	-7.63	0.0	0.12	Temp	-0.99	0.0	0.09	Precision	8.13	0.0	0.22												
RSV	AH	-2519.0	0.68	0.0	Intercept	-7.66	0.0	0.13	AH	-1.12	0.0	0.14	Precision	8.1	0.0	0.24												
RSV	Yt-1/2	-2455.0	0.62	0.0	Intercept	-7.49	0.0	0.13	Yt-1	0.41	0.0	0.11	Yt-2	0.29	0.0	0.12	Precision	7.73	0.0	0.22								
RSV	Temp_Yt-1	-2609.0	0.82	0.71	Intercept	-7.73	0.0	0.1	Temp	-0.78	0.0	0.09	Yt-1	0.35	0.0	0.06	Precision	8.64	0.0	0.22								
RSV	AH_Yt-1	-2625.0	0.84	0.59	Intercept	-7.81	0.0	0.1	AH	-0.94	0.0	0.12	Yt-1	0.37	0.0	0.05	Precision	8.82	0.0	0.22								
RSV	Temp_RH	-2537.0	0.71	0.0	Intercept	-7.63	0.0	0.12	Temp	-0.99	0.0	0.09	RH	0.03	0.52	0.1	Precision	8.14	0.0	0.22								
RSV	AH_RH	-2520.0	0.69	0.0	Intercept	-7.67	0.0	0.14	AH	-1.12	0.0	0.14	RH	0.09	0.05	0.08	Precision	8.13	0.0	0.24								
RSV	Temp_Yt-1/2	-2598.0	0.83	0.11	Intercept	-7.74	0.0	0.1	Temp	-0.75	0.0	0.1	Yt-1	0.27	0.0	0.08	Yt-2	0.12	0.01	0.09	Precision	8.67	0.0	0.22				
RSV	AH_Yt-1/2	-2616.0	0.85	0.21	Intercept	-7.82	0.0	0.1	AH	-0.92	0.0	0.12	Yt-1	0.29	0.0	0.07	Yt-2	0.13	0.0	0.08	Precision	8.87	0.0	0.22				
RSV	Temp_RH_Yt-1	-2609.0	0.82	0.65	Intercept	-7.74	0.0	0.1	Temp	-0.78	0.0	0.09	RH	0.06	0.2	0.08	Yt-1	0.35	0.0	0.05	Precision	8.65	0.0	0.22				
RSV	AH_RH_Yt-1	-2630.0	0.84	0.49	Intercept	-7.83	0.0	0.11	AH	-0.96	0.0	0.12	RH	0.09	0.01	0.07	Yt-1	0.36	0.0	0.05	Precision	8.87	0.0	0.22				
RSV	Temp_RH_Yt-1/2	-2599.0	0.83	0.08	Intercept	-7.75	0.0	0.1	Temp	-0.75	0.0	0.09	RH	0.07	0.09	0.08	Yt-1	0.26	0.0	0.08	Yt-2	0.13	0.0	0.09	Precision	8.7	0.0	0.22
RSV	AH_RH_Yt-1/2	-2623.0	0.85	0.17	Intercept	-7.84	0.0	0.1	AH	-0.93	0.0	0.12	RH	0.11	0.0	0.07	Yt-1	0.27	0.0	0.07	Yt-2	0.14	0.0	0.08	Precision	8.94	0.0	0.22
hCoVs	None	-2473.0	0.0	0.0	Intercept	-7.61	0.0	0.19	Precision	7.07	0.0	0.25																
hCoVs	Yt-1	-2618.0	0.59	0.01	Intercept	-7.92	0.0	0.13	Yt-1	0.63	0.0	0.05	Precision	8.07	0.0	0.22												
hCoVs	Temp	-2640.0	0.6	0.0	Intercept	-7.99	0.0	0.14	Temp	-0.9	0.0	0.11	Precision	8.21	0.0	0.23												
hCoVs	AH	-2620.0	0.56	0.0	Intercept	-7.96	0.0	0.15	AH	-0.93	0.0	0.15	Precision	8.1	0.0	0.24												
hCoVs	Yt-1/2	-2610.0	0.61	0.0	Intercept	-7.94	0.0	0.13	Yt-1	0.43	0.0	0.13	Yt-2	0.25	0.0	0.14	Precision	8.15	0.0	0.23								
hCoVs	Temp_Yt-1	-2742.0	0.79	0.05	Intercept	-8.17	0.0	0.11	Temp	-0.72	0.0	0.1	Yt-1	0.39	0.0	0.05	Precision	8.92	0.0	0.22								
hCoVs	AH_Yt-1	-2755.0	0.81	0.01	Intercept	-8.23	0.0	0.11	AH	-0.84	0.0	0.13	Yt-1	0.43	0.0	0.05	Precision	9.07	0.0	0.22								
hCoVs	Temp_RH	-2647.0	0.62	0.0	Intercept	-8.0	0.0	0.13	Temp	-0.93	0.0	0.11	RH	-0.15	0.0	0.1	Precision	8.25	0.0	0.23								
hCoVs	AH_RH	-2620.0	0.56	0.0	Intercept	-7.96	0.0	0.15	AH	-0.95	0.0	0.15	RH	-0.08	0.11	0.09	Precision	8.11	0.0	0.24								
hCoVs	Temp_Yt-1/2	-2735.0	0.81	0.0	Intercept	-8.19	0.0	0.1	Temp	-0.72	0.0	0.1	Yt-1	0.25	0.0	0.09	Yt-2	0.19	0.0	0.1	Precision	9.01	0.0	0.22				
hCoVs	AH_Yt-1/2	-2744.0	0.82	0.0	Intercept	-8.24	0.0	0.11	AH	-0.83	0.0	0.13	Yt-1	0.32	0.0	0.09	Yt-2	0.14	0.0	0.09	Precision	9.12	0.0	0.22				
hCoVs	Temp_RH_Yt-1	-2746.0	0.8	0.04	Intercept	-8.17	0.0	0.1	Temp	-0.74	0.0	0.1	RH	-0.11	0.01	0.09	Yt-1	0.39	0.0	0.05	Precision	8.95	0.0	0.22				
hCoVs	AH_RH_Yt-1	-2755.0	0.81	0.01	Intercept	-8.23	0.0	0.11	AH	-0.85	0.0	0.12	RH	-0.06	0.14	0.08	Yt-1	0.43	0.0	0.05	Precision	9.08	0.0	0.22				
hCoVs	Temp_RH_Yt-1/2	-2738.0	0.81	0.0	Intercept	-8.18	0.0	0.1	Temp	-0.73	0.0	0.1	RH	-0.09	0.03	0.09	Yt-1	0.26	0.0	0.1	Yt-2	0.17	0.0	0.1	Precision	9.03	0.0	0.22
hCoVs	AH_RH_Yt-1/2	-2743.0	0.82	0.0	Intercept	-8.24	0.0	0.11	AH	-0.84	0.0	0.12	RH	-0.04	0.28	0.08	Yt-1	0.33	0.0	0.09	Yt-2	0.14	0.0	0.1	Precision	9.13	0.0	0.22
IVB	None	-2637.0	0.0	0.0	Intercept	-7.5	0.0	0.25	Precision	6.4	0.0	0.3																
IVB	Yt-1	-2752.0	0.54	0.04	Intercept	-8.01	0.0	0.18	Yt-1	0.7	0.0	0.06	Precision	7.48	0.0	0.25												
IVB	Temp	-2681.0	0.22	0.0	Intercept	-7.61	0.0	0.22	Temp	-0.47	0.0	0.13	Precision	6.69	0.0	0.28												
IVB	AH	-2686.0	0.24	0.0	Intercept	-7.62	0.0	0.22	AH	-0.5	0.0	0.14	Precision	6.71	0.0	0.28												
IVB	Yt-1/2	-2734.0	0.54	0.0	Intercept	-8.0	0.0	0.18	Yt-1	0.65	0.0	0.22	Yt-2	0.06	0.6	0.23	Precision	7.47	0.0	0.25								
IVB	Temp_Yt-1	-2789.0	0.63	0.11	Intercept	-8.12	0.0	0.17	Temp	-0.42	0.0	0.12	Yt-1	0.6	0.0	0.06	Precision	7.76	0.0	0.25								
IVB	AH_Yt-1	-2800.0	0.65	0.04	Intercept	-8.14	0.0	0.17	AH	-0.5	0.0	0.14	Yt-1	0.61	0.0	0.06	Precision	7.84	0.0	0.25								
IVB	Temp_RH	-2686.0	0.25	0.0	Intercept	-7.62	0.0	0.22	Temp	-0.52	0.0	0.13	RH	-0.19	0.01	0.14	Precision	6.73	0.0	0.28								
IVB	AH_RH	-2689.0	0.26	0.0	Intercept	-7.63	0.0	0.22	AH	-0.54	0.0	0.14	RH	-0.15	0.02	0.13	Precision	6.75	0.0	0.28								
IVB	Temp_Yt-1/2	-2773.0	0.63	0.0	Intercept	-8.13	0.0	0.17	Temp	-0.44	0.0	0.13	Yt-1	0.45	0.0	0.19	Yt-2	0.16	0.1	0.19	Precision	7.79	0.0	0.25				
IVB	AH_Yt-1/2	-2783.0	0.65	0.0	Intercept	-8.14	0.0	0.17	AH	-0.5	0.0	0.14	Yt-1	0.53	0.0	0.18	Yt-2	0.08	0.38	0.18	Precision	7.84	0.0	0.25				
IVB	Temp_RH_Yt-1	-2793.0	0.64	0.04	Intercept	-8.12	0.0	0.17	Temp	-0.46	0.0	0.12	RH	-0.16	0.01	0.13	Yt-1	0.59	0.0	0.06	Precision	7.79	0.0	0.25				
IVB	AH_RH_Yt-1	-2803.0	0.66	0.01	Intercept	-8.15	0.0	0.17	AH	-0.53	0.0	0.14	RH	-0.13	0.02	0.12	Yt-1	0.6	0.0	0.05	Precision	7.86	0.0	0.25				
IVB	Temp_RH_Yt-1/2	-2777.0	0.65	0.0	Intercept	-8.13	0.0	0.17	Temp	-0.48	0.0	0.13	RH	-0.16	0.02	0.13	Yt-1	0.46	0.0	0.18	Yt-2	0.14	0.13	0.19	Precision	7.81	0.0	0.25
IVB	AH_RH_Yt-1/2	-2785.0	0.66	0.0	Intercept	-8.15	0.0	0.17	AH	-0.53	0.0	0.14	RH	-0.13	0.03	0.12	Yt-1	0.55	0.0	0.18	Yt-2	0.06	0.52	0.18	Precision	7.86	0.0	0.25
hMPV	None	-2540.0	-0.0	0.0	Intercept	-7.89	0.0	0.16	Precision	7.64	0.0	0.24																
hMPV	Yt-1	-2711.0	0.64	0.01	Intercept	-8.16	0.0	0.11	Yt-1	0.62	0.0	0.05	Precision	8.75	0.0	0.22												
hMPV	Temp	-2626.0	0.38	0.0	Intercept	-8.05	0.0	0.14	Temp	-0.58	0.0	0.11	Precision	8.2	0.0	0.23												
hMPV	AH	-2637.0	0.41	0.0	Intercept	-8.07	0.0	0.14	AH	-0.65	0.0	0.13	Precision	8.27	0.0	0.23												
hMPV	Yt-1/2	-2694.0	0.65	0.0	Intercept	-8.16	0.0	0.11	Yt-1	0.51	0.0	0.22	Yt-2	0.11	0.32	0.22	Precision	8.76	0.0	0.22								
hMPV	Temp_Yt-1	-2756.0	0.72	0.0	Intercept	-8.23	0.0	0.1	Temp	-0.37	0.0	0.1	Yt-1	0.49	0.0	0.06	Precision	9.05	0.0	0.22								
hMPV	AH_Yt-1	-2779.0	0.76	0.01	Intercept	-8.28	0.0	0.1	AH	-0.49	0.0	0.11	Yt-1	0.48	0.0	0.05	Precision	9.22	0.0	0.22								
hMPV	Temp_RH	-2668.0	0.51	0.0	Intercept	-8.1	0.0	0.12	Temp	-0.66	0.0	0.1	RH	-0.36	0.0	0.1	Precision	8.44	0.0	0.22								
hMPV	AH_RH	-2673.0	0.52	0.0	Intercept	-8.13	0.0	0.13	AH	-0.72	0.0	0.12	RH	-0.3	0.0	0.09	Precision	8.49	0.0	0.23								
hMPV	Temp_Yt-1/2	-2742.0	0.73	0.0	Intercept	-8.23	0.0	0.1	Temp	-0.38	0.0	0.1	Yt-1	0.31	0.0	0.16	Yt-2	0.19	0.02	0.16	Precision	9.08	0.0	0.22				
hMPV	AH_Yt-1/2	-2762.0	0.76	0.0	Intercept	-8.27	0.0	0.1	AH	-0.49	0.0	0.11	Yt-1	0.35	0.0	0.16	Yt-2	0.14	0.09	0.15	Precision	9.23	0.0	0.22				
hMPV	Temp_RH_Yt-1	-2787.0	0.77	0.04	Intercept	-8.27	0.0	0.09	Temp	-0.46	0.0	0.09	RH	-0.25	0.0	0.08	Yt-1	0.46	0.0	0.05	Precision	9.25	0.0	0.21				
hMPV	AH_RH_Yt-1	-2804.0	0.79	0.06	Intercept	-8.31	0.0	0.09	AH	-0.55	0.0	0.1	RH	-0.19	0.0	0.07	Yt-1	0.46	0.0	0.05	Precision	9.38	0.0	0.21				
hMPV	Temp_RH_Yt-1/2	-2770.0	0.77	0.0	Intercept	-8.27	0.0	0.09	Temp	-0.47	0.0	0.09	RH	-0.24	0.0	0.08	Yt-1	0.32	0.0	0.16	Yt-2	0.14	0.07	0.16	Precision	9.27	0.0	0.21
hMPV	AH_RH_Yt-1/2	-2786.0	0.79	0.0	Intercept	-8.3	0.0	0.09	AH	-0.55	0.0	0.1	RH	-0.19	0.0	0.07	Yt-1	0.39	0.0	0.15	Yt-2	0.07	0.34	0.15	Precision	9.38	0.0	0.21

USA (2015/16-2020)

Virus	Model	AIC	R^2	pValueAC	Variable1	Coef1	pValue1	error1	Variable2	Coef2	pValue2	error2	Variable3	Coef3	pValue3	error3	Variable4	Coef4	pValue4	error4	Variable5	Coef5	pValue5	error5	Variable6	Coef6	pValue6	error6
IVA	None	-2488.0	0.0	0.0	Intercept	-6.07	0.0	0.19	Precision	5.25	0.0	0.24																
IVA	Yt-1	-2748.0	0.7	0.0	Intercept	-6.64	0.0	0.12	Yt-1	0.86	0.0	0.05	Precision	6.73	0.0	0.2												
IVA	Temp	-2622.0	0.44	0.0	Intercept	-6.35	0.0	0.16	Temp	-0.77	0.0	0.12	Precision	5.99	0.0	0.23												
IVA	AH	-2602.0	0.39	0.0	Intercept	-6.3	0.0	0.16	AH	-0.74	0.0	0.13	Precision	5.88	0.0	0.23												
IVA	Yt-1/2	-2738.0	0.71	0.0	Intercept	-6.65	0.0	0.12	Yt-1	1.19	0.0	0.25	Yt-2	-0.34	0.01	0.25	Precision	6.76	0.0	0.2								
IVA	Temp_Yt-1	-2851.0	0.81	0.08	Intercept	-6.8	0.0	0.1	Temp	-0.59	0.0	0.1	Yt-1	0.64	0.0	0.05	Precision	7.29	0.0	0.2								
IVA	AH_Yt-1	-2857.0	0.81	0.01	Intercept	-6.84	0.0	0.11	AH	-0.66	0.0	0.11	Yt-1	0.67	0.0	0.05	Precision	7.36	0.0	0.2								
IVA	Temp_RH	-2621.0	0.45	0.0	Intercept	-6.35	0.0	0.16	Temp	-0.79	0.0	0.12	RH	-0.06	0.24	0.1	Precision	6.0	0.0	0.23								
IVA	AH_RH	-2601.0	0.4	0.0	Intercept	-6.31	0.0	0.16	AH	-0.74	0.0	0.13	RH	0.07	0.19	0.1	Precision	5.88	0.0	0.23								
IVA	Temp_Yt-1/2	-2835.0	0.81	0.09	Intercept	-6.79	0.0	0.1	Temp	-0.58	0.0	0.1	Yt-1	0.68	0.0	0.19	Yt-2	-0.04	0.69	0.18	Precision	7.28	0.0	0.2				
IVA	AH_Yt-1/2	-2844.0	0.82	0.07	Intercept	-6.83	0.0	0.11	AH	-0.64	0.0	0.11	Yt-1	0.83	0.0	0.18	Yt-2	-0.16	0.07	0.18	Precision	7.36	0.0	0.2				
IVA	Temp_RH_Yt-1	-2850.0	0.81	0.01	Intercept	-6.8	0.0	0.11	Temp	-0.59	0.0	0.1	RH	0.03	0.3	0.06	Yt-1	0.64	0.0	0.05	Precision	7.3	0.0	0.2				
IVA	AH_RH_Yt-1	-2866.0	0.82	0.0	Intercept	-6.87	0.0	0.11	AH	-0.7	0.0	0.11	RH	0.1	0.0	0.06	Yt-1	0.66	0.0	0.05	Precision	7.43	0.0	0.2				
IVA	Temp_RH_Yt-1/2	-2834.0	0.81	0.0	Intercept	-6.8	0.0	0.1	Temp	-0.58	0.0	0.1	RH	0.03	0.3	0.06	Yt-1	0.69	0.0	0.19	Yt-2	-0.04	0.64	0.18	Precision	7.29	0.0	0.2
IVA	AH_RH_Yt-1/2	-2853.0	0.83	0.0	Intercept	-6.86	0.0	0.11	AH	-0.68	0.0	0.11	RH	0.1	0.0	0.06	Yt-1	0.8	0.0	0.18	Yt-2	-0.15	0.1	0.17	Precision	7.44	0.0	0.2
RSV	None	-1844.0	0.0	0.0	Intercept	-6.16	0.0	0.19	Precision	5.62	0.0	0.26																
RSV	Yt-1	-2043.0	0.71	0.0	Intercept	-6.52	0.0	0.12	Yt-1	0.76	0.0	0.06	Precision	6.97	0.0	0.22												
RSV	Temp	-2121.0	0.8	0.01	Intercept	-6.67	0.0	0.11	Temp	-1.09	0.0	0.08	Precision	7.45	0.0	0.22												
RSV	AH	-2063.0	0.72	0.0	Intercept	-6.65	0.0	0.13	AH	-1.13	0.0	0.13	Precision	7.17	0.0	0.24												
RSV	Yt-1/2	-2028.0	0.71	0.0	Intercept	-6.52	0.0	0.12	Yt-1	0.66	0.0	0.17	Yt-2	0.12	0.17	0.18	Precision	6.99	0.0	0.22								
RSV	Temp_Yt-1	-2225.0	0.9	0.97	Intercept	-6.79	0.0	0.08	Temp	-0.77	0.0	0.08	Yt-1	0.46	0.0	0.06	Precision	8.18	0.0	0.21								
RSV	AH_Yt-1	-2227.0	0.9	0.86	Intercept	-6.85	0.0	0.08	AH	-0.86	0.0	0.1	Yt-1	0.51	0.0	0.05	Precision	8.27	0.0	0.22								
RSV	Temp_RH	-2149.0	0.83	0.0	Intercept	-6.73	0.0	0.1	Temp	-1.11	0.0	0.08	RH	0.18	0.0	0.06	Precision	7.66	0.0	0.22								
RSV	AH_RH	-2149.0	0.83	0.0	Intercept	-6.83	0.0	0.11	AH	-1.32	0.0	0.12	RH	0.32	0.0	0.06	Precision	7.8	0.0	0.23								
RSV	Temp_Yt-1/2	-2207.0	0.9	0.98	Intercept	-6.78	0.0	0.08	Temp	-0.76	0.0	0.08	Yt-1	0.48	0.0	0.12	Yt-2	-0.02	0.78	0.12	Precision	8.18	0.0	0.22				
RSV	AH_Yt-1/2	-2211.0	0.9	0.88	Intercept	-6.84	0.0	0.08	AH	-0.86	0.0	0.1	Yt-1	0.58	0.0	0.11	Yt-2	-0.08	0.17	0.12	Precision	8.28	0.0	0.22				
RSV	Temp_RH_Yt-1	-2250.0	0.91	0.73	Intercept	-6.84	0.0	0.08	Temp	-0.83	0.0	0.08	RH	0.12	0.0	0.05	Yt-1	0.42	0.0	0.06	Precision	8.37	0.0	0.21				
RSV	AH_RH_Yt-1	-2321.0	0.94	0.89	Intercept	-6.98	0.0	0.07	AH	-1.08	0.0	0.09	RH	0.22	0.0	0.04	Yt-1	0.42	0.0	0.04	Precision	8.9	0.0	0.21				
RSV	Temp_RH_Yt-1/2	-2231.0	0.91	0.67	Intercept	-6.82	0.0	0.08	Temp	-0.82	0.0	0.08	RH	0.12	0.0	0.04	Yt-1	0.43	0.0	0.11	Yt-2	-0.01	0.85	0.11	Precision	8.36	0.0	0.21
RSV	AH_RH_Yt-1/2	-2302.0	0.94	0.9	Intercept	-6.97	0.0	0.07	AH	-1.08	0.0	0.09	RH	0.21	0.0	0.04	Yt-1	0.45	0.0	0.09	Yt-2	-0.04	0.42	0.09	Precision	8.9	0.0	0.22
hCoVs	None	-2817.0	0.0	0.0	Intercept	-8.16	0.0	0.19	Precision	7.58	0.0	0.25																
hCoVs	Yt-1	-3069.0	0.76	0.0	Intercept	-8.67	0.0	0.11	Yt-1	0.88	0.0	0.06	Precision	9.21	0.0	0.21												
hCoVs	Temp	-3042.0	0.69	0.0	Intercept	-8.67	0.0	0.13	Temp	-1.05	0.0	0.1	Precision	9.05	0.0	0.22												
hCoVs	AH	-2983.0	0.58	0.0	Intercept	-8.56	0.0	0.14	AH	-0.97	0.0	0.14	Precision	8.71	0.0	0.24												
hCoVs	Yt-1/2	-3049.0	0.76	0.0	Intercept	-8.66	0.0	0.11	Yt-1	0.86	0.0	0.18	Yt-2	0.02	0.86	0.19	Precision	9.21	0.0	0.21								
hCoVs	Temp_Yt-1	-3162.0	0.85	0.99	Intercept	-8.78	0.0	0.09	Temp	-0.63	0.0	0.11	Yt-1	0.51	0.0	0.08	Precision	9.76	0.0	0.21								
hCoVs	AH_Yt-1	-3177.0	0.86	0.97	Intercept	-8.84	0.0	0.09	AH	-0.67	0.0	0.11	Yt-1	0.58	0.0	0.06	Precision	9.9	0.0	0.21								
hCoVs	Temp_RH	-3046.0	0.7	0.0	Intercept	-8.69	0.0	0.13	Temp	-1.05	0.0	0.1	RH	0.09	0.01	0.07	Precision	9.1	0.0	0.23								
hCoVs	AH_RH	-3008.0	0.64	0.0	Intercept	-8.63	0.0	0.14	AH	-1.03	0.0	0.13	RH	0.22	0.0	0.08	Precision	8.89	0.0	0.24								
hCoVs	Temp_Yt-1/2	-3145.0	0.85	0.97	Intercept	-8.78	0.0	0.09	Temp	-0.65	0.0	0.11	Yt-1	0.38	0.0	0.16	Yt-2	0.13	0.07	0.14	Precision	9.78	0.0	0.21				
hCoVs	AH_Yt-1/2	-3157.0	0.86	0.96	Intercept	-8.83	0.0	0.09	AH	-0.67	0.0	0.11	Yt-1	0.55	0.0	0.14	Yt-2	0.04	0.61	0.14	Precision	9.9	0.0	0.21				
hCoVs	Temp_RH_Yt-1	-3183.0	0.87	0.27	Intercept	-8.83	0.0	0.09	Temp	-0.66	0.0	0.1	RH	0.13	0.0	0.05	Yt-1	0.51	0.0	0.07	Precision	9.92	0.0	0.21				
hCoVs	AH_RH_Yt-1	-3230.0	0.9	0.33	Intercept	-8.94	0.0	0.09	AH	-0.81	0.0	0.1	RH	0.18	0.0	0.04	Yt-1	0.55	0.0	0.05	Precision	10.27	0.0	0.21				
hCoVs	Temp_RH_Yt-1/2	-3165.0	0.87	0.02	Intercept	-8.83	0.0	0.09	Temp	-0.68	0.0	0.1	RH	0.12	0.0	0.05	Yt-1	0.41	0.0	0.15	Yt-2	0.1	0.16	0.13	Precision	9.93	0.0	0.21
hCoVs	AH_RH_Yt-1/2	-3211.0	0.9	0.04	Intercept	-8.94	0.0	0.09	AH	-0.82	0.0	0.1	RH	0.18	0.0	0.04	Yt-1	0.52	0.0	0.12	Yt-2	0.03	0.62	0.11	Precision	10.27	0.0	0.21
IVB	None	-2807.0	0.0	0.0	Intercept	-6.86	0.0	0.18	Precision	6.17	0.0	0.24																
IVB	Yt-1	-2973.0	0.55	0.66	Intercept	-7.2	0.0	0.13	Yt-1	0.59	0.0	0.04	Precision	7.11	0.0	0.21												
IVB	Temp	-2859.0	0.21	0.0	Intercept	-6.96	0.0	0.17	Temp	-0.43	0.0	0.11	Precision	6.44	0.0	0.23												
IVB	AH	-2866.0	0.23	0.0	Intercept	-6.97	0.0	0.16	AH	-0.48	0.0	0.12	Precision	6.48	0.0	0.23												
IVB	Yt-1/2	-2964.0	0.57	0.0	Intercept	-7.23	0.0	0.13	Yt-1	0.46	0.0	0.1	Yt-2	0.17	0.0	0.11	Precision	7.16	0.0	0.21								
IVB	Temp_Yt-1	-3037.0	0.66	0.44	Intercept	-7.34	0.0	0.12	Temp	-0.44	0.0	0.1	Yt-1	0.53	0.0	0.04	Precision	7.49	0.0	0.2								
IVB	AH_Yt-1	-3051.0	0.68	0.48	Intercept	-7.37	0.0	0.12	AH	-0.51	0.0	0.11	Yt-1	0.53	0.0	0.04	Precision	7.58	0.0	0.2								
IVB	Temp_RH	-2868.0	0.25	0.0	Intercept	-6.98	0.0	0.16	Temp	-0.47	0.0	0.11	RH	-0.19	0.0	0.11	Precision	6.5	0.0	0.23								
IVB	AH_RH	-2868.0	0.25	0.0	Intercept	-6.98	0.0	0.16	AH	-0.48	0.0	0.12	RH	-0.11	0.05	0.11	Precision	6.5	0.0	0.23								
IVB	Temp_Yt-1/2	-3024.0	0.67	0.01	Intercept	-7.35	0.0	0.12	Temp	-0.43	0.0	0.1	Yt-1	0.44	0.0	0.09	Yt-2	0.11	0.03	0.1	Precision	7.52	0.0	0.2				
IVB	AH_Yt-1/2	-3037.0	0.69	0.02	Intercept	-7.38	0.0	0.12	AH	-0.5	0.0	0.11	Yt-1	0.46	0.0	0.09	Yt-2	0.09	0.07	0.1	Precision	7.6	0.0	0.21				
IVB	Temp_RH_Yt-1	-3060.0	0.7	0.38	Intercept	-7.38	0.0	0.12	Temp	-0.47	0.0	0.09	RH	-0.21	0.0	0.08	Yt-1	0.56	0.0	0.04	Precision	7.62	0.0	0.2				
IVB	AH_RH_Yt-1	-3061.0	0.7	0.37	Intercept	-7.39	0.0	0.12	AH	-0.5	0.0	0.11	RH	-0.14	0.0	0.08	Yt-1	0.56	0.0	0.04	Precision	7.64	0.0	0.2				
IVB	Temp_RH_Yt-1/2	-3043.0	0.7	0.24	Intercept	-7.38	0.0	0.12	Temp	-0.47	0.0	0.1	RH	-0.21	0.0	0.09	Yt-1	0.54	0.0	0.1	Yt-2	0.02	0.64	0.1	Precision	7.63	0.0	0.2
IVB	AH_RH_Yt-1/2	-3045.0	0.7	0.15	Intercept	-7.39	0.0	0.12	AH	-0.5	0.0	0.11	RH	-0.14	0.0	0.08	Yt-1	0.53	0.0	0.1	Yt-2	0.04	0.49	0.1	Precision	7.65	0.0	0.2
hMPV	None	-2095.0	0.0	0.0	Intercept	-6.99	0.0	0.17	Precision	6.68	0.0	0.25																
hMPV	Yt-1	-2311.0	0.76	0.0	Intercept	-7.36	0.0	0.1	Yt-1	0.77	0.0	0.05	Precision	8.21	0.0	0.22												
hMPV	Temp	-2212.0	0.5	0.0	Intercept	-7.22	0.0	0.14	Temp	-0.69	0.0	0.11	Precision	7.47	0.0	0.23												
hMPV	AH	-2215.0	0.5	0.0	Intercept	-7.23	0.0	0.14	AH	-0.75	0.0	0.13	Precision	7.5	0.0	0.24												
hMPV	Yt-1/2	-2281.0	0.76	0.0	Intercept	-7.36	0.0	0.1	Yt-1	0.84	0.0	0.23	Yt-2	-0.07	0.55	0.23	Precision	8.21	0.0	0.22								
hMPV	Temp_Yt-1	-2404.0	0.86	0.01	Intercept	-7.5	0.0	0.08	Temp	-0.49	0.0	0.08	Yt-1	0.61	0.0	0.05	Precision	8.85	0.0	0.22								
hMPV	AH_Yt-1	-2425.0	0.88	0.0	Intercept	-7.54	0.0	0.08	AH	-0.58	0.0	0.09	Yt-1	0.6	0.0	0.05	Precision	9.01	0.0	0.22								
hMPV	Temp_RH	-2218.0	0.52	0.0	Intercept	-7.23	0.0	0.13	Temp	-0.71	0.0	0.11	RH	-0.15	0.0	0.1	Precision	7.52	0.0	0.23								
hMPV	AH_RH	-2214.0	0.51	0.0	Intercept	-7.23	0.0	0.14	AH	-0.75	0.0	0.13	RH	-0.06	0.27	0.1	Precision	7.51	0.0	0.24								
hMPV	Temp_Yt-1/2	-2376.0	0.86	0.0	Intercept	-7.5	0.0	0.08	Temp	-0.51	0.0	0.09	Yt-1	0.47	0.0	0.18	Yt-2	0.14	0.11	0.17	Precision	8.87	0.0	0.22				
hMPV	AH_Yt-1/2	-2393.0	0.88	0.0	Intercept	-7.54	0.0	0.08	AH	-0.58	0.0	0.09	Yt-1	0.58	0.0	0.16	Yt-2	0.02	0.75	0.15	Precision	9.0	0.0	0.22				
hMPV	Temp_RH_Yt-1	-2407.0	0.86	0.01	Intercept	-7.5	0.0	0.08	Temp	-0.49	0.0	0.08	RH	-0.06	0.02	0.05	Yt-1	0.61	0.0	0.05	Precision	8.88	0.0	0.22				
hMPV	AH_RH_Yt-1	-2423.0	0.88	0.0	Intercept	-7.54	0.0	0.08	AH	-0.58	0.0	0.09	RH	0.01	0.67	0.05	Yt-1	0.6	0.0	0.05	Precision	9.01	0.0	0.22				
hMPV	Temp_RH_Yt-1/2	-2377.0	0.87	0.0	Intercept	-7.5	0.0	0.08	Temp	-0.5	0.0	0.08	RH	-0.05	0.06	0.05	Yt-1	0.51	0.0	0.18	Yt-2	0.1	0.26	0.17	Precision	8.89	0.0	0.22
hMPV	AH_RH_Yt-1/2	-2392.0	0.88	0.0	Intercept	-7.54	0.0	0.08	AH	-0.59	0.0	0.1	RH	0.02	0.57	0.05	Yt-1	0.57	0.0	0.17	Yt-2	0.04	0.63	0.16	Precision	9.01	0.0	0.22
