# Supplementary material for: The effects of weather and mobility on respiratory viruses dynamics before and during the COVID-19 pandemic in the USA and Canada
Source: PLOS Digit Health. 2023 Dec 21;2(12):e0000405. doi: 10.1371/journal.pdig.0000405 (PMC10734953; doi:10.1371/journal.pdig.0000405)
Supplement: S2 Table — (PDF) [file pdig.0000405.s015.pdf]

S2 Table. Regression models results for the weather analysis for Canada and the USA in the pandemic period (2020-2023).

pValueAC, p-value autocorrelation(AC) Breusch-Godfrey test up to 53 weeks; Coef, regression coefficient; Temp, temperature; AH, absolute humidity; RH, relative humidity; Yt-1, AC term 1 week; Yt-1/2 AC term 1 and 2 weeks. Models used for comparison in grey.

CANADA (2020-2023)

Virus	Model	AIC	R^2	pValueAC	Variable1	Coef1	pValue1	error1	Variable2	Coef2	pValue2	error2	Variable3	Coef3	pValue3	error3	Variable4	Coef4	pValue4	error4	Variable5	Coef5	pValue5	error5	Variable6	Coef6	pValue6	error6
IVA	None	-2916.0	-0.0	0.0	Intercept	-7.79	0.0	0.41	Precision	5.92	0.0	0.44																
IVA	Yt-1	-2991.0	0.4	0.0	Intercept	-8.45	0.0	0.32	Yt-1	0.7	0.0	0.07	Precision	6.91	0.0	0.37												
IVA	Temp	-2916.0	0.01	0.0	Intercept	-7.79	0.0	0.4	Temp	-0.12	0.14	0.15	Precision	5.94	0.0	0.44												
IVA	AH	-2916.0	0.02	0.0	Intercept	-7.79	0.0	0.4	AH	-0.12	0.13	0.15	Precision	5.94	0.0	0.44												
IVA	Yt-1/2	-2989.0	0.4	0.0	Intercept	-8.45	0.0	0.32	Yt-1	0.7	0.0	0.34	Yt-2	-0.01	0.97	0.36	Precision	6.92	0.0	0.37								
IVA	Temp_Yt-1	-2990.0	0.41	0.0	Intercept	-8.45	0.0	0.32	Temp	-0.06	0.49	0.16	Yt-1	0.69	0.0	0.08	Precision	6.92	0.0	0.37								
IVA	AH_Yt-1	-2990.0	0.41	0.0	Intercept	-8.46	0.0	0.32	AH	-0.07	0.35	0.15	Yt-1	0.68	0.0	0.08	Precision	6.93	0.0	0.37								
IVA	Temp_RH	-2915.0	0.02	0.0	Intercept	-7.8	0.0	0.4	Temp	-0.1	0.19	0.15	RH	0.07	0.36	0.16	Precision	5.94	0.0	0.44								
IVA	AH_RH	-2915.0	0.02	0.0	Intercept	-7.8	0.0	0.4	AH	-0.12	0.13	0.15	RH	0.09	0.26	0.15	Precision	5.95	0.0	0.44								
IVA	Temp_Yt-1/2	-2988.0	0.41	0.0	Intercept	-8.45	0.0	0.32	Temp	-0.06	0.48	0.16	Yt-1	0.7	0.0	0.34	Yt-2	-0.02	0.92	0.36	Precision	6.92	0.0	0.37				
IVA	AH_Yt-1/2	-2988.0	0.41	0.0	Intercept	-8.46	0.0	0.32	AH	-0.07	0.34	0.16	Yt-1	0.7	0.0	0.33	Yt-2	-0.02	0.92	0.35	Precision	6.93	0.0	0.37				
IVA	Temp_RH_Yt-1	-2988.0	0.41	0.0	Intercept	-8.45	0.0	0.32	Temp	-0.05	0.54	0.16	RH	0.04	0.63	0.16	Yt-1	0.68	0.0	0.08	Precision	6.92	0.0	0.37				
IVA	AH_RH_Yt-1	-2988.0	0.41	0.0	Intercept	-8.46	0.0	0.32	AH	-0.07	0.35	0.15	RH	0.04	0.57	0.15	Yt-1	0.68	0.0	0.08	Precision	6.93	0.0	0.37				
IVA	Temp_RH_Yt-1/2	-2986.0	0.41	0.0	Intercept	-8.46	0.0	0.32	Temp	-0.05	0.54	0.16	RH	0.04	0.62	0.16	Yt-1	0.71	0.0	0.33	Yt-2	-0.03	0.86	0.36	Precision	6.92	0.0	0.37
IVA	AH_RH_Yt-1/2	-2986.0	0.41	0.0	Intercept	-8.46	0.0	0.32	AH	-0.07	0.34	0.15	RH	0.05	0.55	0.16	Yt-1	0.71	0.0	0.33	Yt-2	-0.03	0.85	0.35	Precision	6.93	0.0	0.37
RSV	None	-2327.0	0.0	0.0	Intercept	-7.92	0.0	0.29	Precision	6.72	0.0	0.34																
RSV	Yt-1	-2467.0	0.61	0.0	Intercept	-8.5	0.0	0.2	Yt-1	0.84	0.0	0.08	Precision	7.97	0.0	0.28												
RSV	Temp	-2330.0	0.03	0.0	Intercept	-7.94	0.0	0.29	Temp	-0.16	0.03	0.15	Precision	6.76	0.0	0.34												
RSV	AH	-2329.0	0.03	0.0	Intercept	-7.93	0.0	0.29	AH	-0.15	0.05	0.15	Precision	6.75	0.0	0.34												
RSV	Yt-1/2	-2465.0	0.61	0.0	Intercept	-8.5	0.0	0.2	Yt-1	0.8	0.0	0.38	Yt-2	0.04	0.86	0.38	Precision	7.97	0.0	0.28								
RSV	Temp_Yt-1	-2465.0	0.61	0.0	Intercept	-8.5	0.0	0.2	Temp	0.02	0.76	0.15	Yt-1	0.84	0.0	0.09	Precision	7.97	0.0	0.28								
RSV	AH_Yt-1	-2465.0	0.61	0.0	Intercept	-8.5	0.0	0.2	AH	0.01	0.92	0.15	Yt-1	0.84	0.0	0.09	Precision	7.97	0.0	0.28								
RSV	Temp_RH	-2331.0	0.05	0.0	Intercept	-7.95	0.0	0.29	Temp	-0.13	0.08	0.15	RH	0.13	0.08	0.15	Precision	6.78	0.0	0.34								
RSV	AH_RH	-2331.0	0.05	0.0	Intercept	-7.95	0.0	0.28	AH	-0.14	0.07	0.15	RH	0.15	0.05	0.15	Precision	6.78	0.0	0.34								
RSV	Temp_Yt-1/2	-2463.0	0.61	0.0	Intercept	-8.5	0.0	0.2	Temp	0.03	0.73	0.15	Yt-1	0.8	0.0	0.38	Yt-2	0.05	0.82	0.39	Precision	7.97	0.0	0.28				
RSV	AH_Yt-1/2	-2463.0	0.61	0.0	Intercept	-8.5	0.0	0.2	AH	0.01	0.9	0.15	Yt-1	0.8	0.0	0.38	Yt-2	0.04	0.85	0.39	Precision	7.97	0.0	0.28				
RSV	Temp_RH_Yt-1	-2463.0	0.61	0.0	Intercept	-8.5	0.0	0.2	Temp	0.02	0.83	0.15	RH	-0.04	0.59	0.14	Yt-1	0.86	0.0	0.1	Precision	7.97	0.0	0.28				
RSV	AH_RH_Yt-1	-2463.0	0.61	0.0	Intercept	-8.5	0.0	0.2	AH	0.01	0.93	0.15	RH	-0.04	0.56	0.14	Yt-1	0.85	0.0	0.1	Precision	7.97	0.0	0.28				
RSV	Temp_RH_Yt-1/2	-2461.0	0.61	0.0	Intercept	-8.5	0.0	0.2	Temp	0.02	0.8	0.15	RH	-0.04	0.58	0.14	Yt-1	0.81	0.0	0.38	Yt-2	0.05	0.8	0.39	Precision	7.97	0.0	0.28
RSV	AH_RH_Yt-1/2	-2461.0	0.61	0.0	Intercept	-8.5	0.0	0.2	AH	0.01	0.9	0.15	RH	-0.04	0.55	0.14	Yt-1	0.81	0.0	0.38	Yt-2	0.05	0.81	0.39	Precision	7.97	0.0	0.28
hCoVs	None	-2365.0	-0.0	0.0	Intercept	-8.33	0.0	0.27	Precision	7.3	0.0	0.32																
hCoVs	Yt-1	-2484.0	0.55	0.67	Intercept	-8.71	0.0	0.19	Yt-1	0.69	0.0	0.07	Precision	8.26	0.0	0.27												
hCoVs	Temp	-2375.0	0.08	0.0	Intercept	-8.36	0.0	0.26	Temp	-0.27	0.0	0.15	Precision	7.39	0.0	0.32												
hCoVs	AH	-2374.0	0.07	0.0	Intercept	-8.36	0.0	0.26	AH	-0.25	0.0	0.16	Precision	7.38	0.0	0.32												
hCoVs	Yt-1/2	-2484.0	0.56	0.03	Intercept	-8.73	0.0	0.19	Yt-1	0.54	0.0	0.19	Yt-2	0.18	0.08	0.21	Precision	8.29	0.0	0.27								
hCoVs	Temp_Yt-1	-2482.0	0.55	0.03	Intercept	-8.72	0.0	0.19	Temp	-0.04	0.62	0.15	Yt-1	0.68	0.0	0.09	Precision	8.27	0.0	0.27								
hCoVs	AH_Yt-1	-2484.0	0.56	0.07	Intercept	-8.73	0.0	0.19	AH	-0.1	0.18	0.15	Yt-1	0.67	0.0	0.08	Precision	8.29	0.0	0.27								
hCoVs	Temp_RH	-2373.0	0.08	0.0	Intercept	-8.36	0.0	0.26	Temp	-0.28	0.0	0.15	RH	-0.04	0.59	0.14	Precision	7.39	0.0	0.32								
hCoVs	AH_RH	-2372.0	0.07	0.0	Intercept	-8.36	0.0	0.26	AH	-0.26	0.0	0.16	RH	-0.01	0.93	0.14	Precision	7.38	0.0	0.32								
hCoVs	Temp_Yt-1/2	-2482.0	0.56	0.0	Intercept	-8.73	0.0	0.19	Temp	-0.02	0.83	0.15	Yt-1	0.54	0.0	0.19	Yt-2	0.18	0.1	0.21	Precision	8.29	0.0	0.27				
hCoVs	AH_Yt-1/2	-2484.0	0.56	0.01	Intercept	-8.74	0.0	0.19	AH	-0.09	0.25	0.15	Yt-1	0.53	0.0	0.19	Yt-2	0.16	0.12	0.21	Precision	8.31	0.0	0.27				
hCoVs	Temp_RH_Yt-1	-2481.0	0.55	0.02	Intercept	-8.71	0.0	0.19	Temp	-0.05	0.53	0.16	RH	-0.04	0.53	0.13	Yt-1	0.68	0.0	0.09	Precision	8.27	0.0	0.27				
hCoVs	AH_RH_Yt-1	-2482.0	0.56	0.04	Intercept	-8.73	0.0	0.19	AH	-0.11	0.17	0.15	RH	-0.04	0.54	0.13	Yt-1	0.67	0.0	0.08	Precision	8.29	0.0	0.27				
hCoVs	Temp_RH_Yt-1/2	-2481.0	0.56	0.0	Intercept	-8.73	0.0	0.19	Temp	-0.03	0.72	0.16	RH	-0.05	0.5	0.13	Yt-1	0.54	0.0	0.19	Yt-2	0.18	0.09	0.21	Precision	8.29	0.0	0.27
hCoVs	AH_RH_Yt-1/2	-2482.0	0.57	0.0	Intercept	-8.74	0.0	0.19	AH	-0.09	0.23	0.15	RH	-0.05	0.48	0.13	Yt-1	0.53	0.0	0.19	Yt-2	0.17	0.11	0.21	Precision	8.31	0.0	0.27
IVB	None	-3583.0	0.0	1.0	Intercept	-11.51	0.0	0.33	Precision	10.05	0.0	0.38																
IVB	Yt-1	-3678.0	0.48	1.0	Intercept	-12.5	0.0	0.27	Yt-1	0.57	0.0	0.04	Precision	11.46	0.0	0.33												
IVB	Temp	-3582.0	0.01	1.0	Intercept	-11.51	0.0	0.33	Temp	-0.07	0.38	0.15	Precision	10.05	0.0	0.37												
IVB	AH	-3582.0	0.01	1.0	Intercept	-11.51	0.0	0.33	AH	-0.09	0.26	0.15	Precision	10.05	0.0	0.37												
IVB	Yt-1/2	-3695.0	0.54	0.0	Intercept	-12.67	0.0	0.26	Yt-1	-0.05	0.49	0.14	Yt-2	0.7	0.0	0.16	Precision	11.71	0.0	0.32								
IVB	Temp_Yt-1	-3676.0	0.48	1.0	Intercept	-12.5	0.0	0.27	Temp	-0.06	0.45	0.14	Yt-1	0.56	0.0	0.04	Precision	11.46	0.0	0.33								
IVB	AH_Yt-1	-3677.0	0.48	1.0	Intercept	-12.51	0.0	0.27	AH	-0.08	0.26	0.15	Yt-1	0.56	0.0	0.04	Precision	11.47	0.0	0.33								
IVB	Temp_RH	-3580.0	0.01	1.0	Intercept	-11.51	0.0	0.33	Temp	-0.06	0.44	0.15	RH	0.04	0.63	0.17	Precision	10.05	0.0	0.37								
IVB	AH_RH	-3580.0	0.01	1.0	Intercept	-11.51	0.0	0.33	AH	-0.09	0.27	0.15	RH	0.05	0.57	0.16	Precision	10.06	0.0	0.37								
IVB	Temp_Yt-1/2	-3693.0	0.54	0.0	Intercept	-12.68	0.0	0.26	Temp	-0.05	0.46	0.14	Yt-1	-0.05	0.49	0.14	Yt-2	0.69	0.0	0.16	Precision	11.72	0.0	0.32				
IVB	AH_Yt-1/2	-3694.0	0.54	0.0	Intercept	-12.68	0.0	0.26	AH	-0.08	0.27	0.15	Yt-1	-0.05	0.52	0.14	Yt-2	0.69	0.0	0.16	Precision	11.73	0.0	0.32				
IVB	Temp_RH_Yt-1	-3676.0	0.48	1.0	Intercept	-12.5	0.0	0.27	Temp	-0.04	0.54	0.14	RH	0.1	0.22	0.16	Yt-1	0.57	0.0	0.04	Precision	11.47	0.0	0.32				
IVB	AH_RH_Yt-1	-3677.0	0.49	1.0	Intercept	-12.51	0.0	0.27	AH	-0.08	0.26	0.15	RH	0.1	0.19	0.16	Yt-1	0.57	0.0	0.04	Precision	11.48	0.0	0.32				
IVB	Temp_RH_Yt-1/2	-3693.0	0.54	0.0	Intercept	-12.67	0.0	0.26	Temp	-0.05	0.54	0.14	RH	0.09	0.26	0.16	Yt-1	-0.05	0.54	0.14	Yt-2	0.7	0.0	0.16	Precision	11.72	0.0	0.32
IVB	AH_RH_Yt-1/2	-3694.0	0.55	0.0	Intercept	-12.67	0.0	0.26	AH	-0.08	0.26	0.14	RH	0.1	0.22	0.16	Yt-1	-0.04	0.57	0.14	Yt-2	0.69	0.0	0.16	Precision	11.73	0.0	0.32
hMPV	None	-2548.0	0.0	0.0	Intercept	-8.76	0.0	0.28	Precision	7.62	0.0	0.34																
hMPV	Yt-1	-2656.0	0.52	0.17	Intercept	-9.12	0.0	0.21	Yt-1	0.68	0.0	0.08	Precision	8.49	0.0	0.28												
hMPV	Temp	-2548.0	0.02	0.0	Intercept	-8.77	0.0	0.28	Temp	-0.12	0.12	0.15	Precision	7.64	0.0	0.33												
hMPV	AH	-2548.0	0.01	0.0	Intercept	-8.77	0.0	0.28	AH	-0.11	0.14	0.15	Precision	7.64	0.0	0.33												
hMPV	Yt-1/2	-2677.0	0.59	0.05	Intercept	-9.23	0.0	0.2	Yt-1	0.47	0.0	0.12	Yt-2	0.37	0.0	0.13	Precision	8.73	0.0	0.28								
hMPV	Temp_Yt-1	-2660.0	0.54	0.26	Intercept	-9.14	0.0	0.2	Temp	-0.15	0.02	0.13	Yt-1	0.7	0.0	0.08	Precision	8.54	0.0	0.28								
hMPV	AH_Yt-1	-2660.0	0.54	0.26	Intercept	-9.14	0.0	0.2	AH	-0.15	0.02	0.13	Yt-1	0.7	0.0	0.08	Precision	8.55	0.0	0.28								
hMPV	Temp_RH	-2547.0	0.02	0.0	Intercept	-8.77	0.0	0.28	Temp	-0.11	0.17	0.15	RH	0.05	0.51	0.15	Precision	7.64	0.0	0.33								
hMPV	AH_RH	-2547.0	0.02	0.0	Intercept	-8.77	0.0	0.28	AH	-0.11	0.16	0.15	RH	0.06	0.39	0.15	Precision	7.64	0.0	0.33								
hMPV	Temp_Yt-1/2	-2678.0	0.6	0.04	Intercept	-9.23	0.0	0.19	Temp	-0.09	0.13	0.12	Yt-1	0.47	0.0	0.13	Yt-2	0.37	0.0	0.13	Precision	8.74	0.0	0.27				
hMPV	AH_Yt-1/2	-2678.0	0.6	0.04	Intercept	-9.23	0.0	0.19	AH	-0.09	0.12	0.12	Yt-1	0.47	0.0	0.13	Yt-2	0.37	0.0	0.14	Precision	8.75	0.0	0.27				
hMPV	Temp_RH_Yt-1	-2662.0	0.55	0.23	Intercept	-9.16	0.0	0.2	Temp	-0.12	0.07	0.13	RH	0.14	0.04	0.14	Yt-1	0.73	0.0	0.08	Precision	8.59	0.0	0.28				
hMPV	AH_RH_Yt-1	-2663.0	0.55	0.21	Intercept	-9.17	0.0	0.2	AH	-0.13	0.04	0.13	RH	0.15	0.02	0.13	Yt-1	0.73	0.0	0.08	Precision	8.6	0.0	0.28				
hMPV	Temp_RH_Yt-1/2	-2681.0	0.61	0.01	Intercept	-9.27	0.0	0.19	Temp	-0.06	0.35	0.12	RH	0.16	0.02	0.13	Yt-1	0.51	0.0	0.12	Yt-2	0.37	0.0	0.13	Precision	8.82	0.0	0.28
hMPV	AH_RH_Yt-1/2	-2682.0	0.61	0.01	Intercept	-9.27	0.0	0.19	AH	-0.07	0.21	0.12	RH	0.16	0.02	0.13	Yt-1	0.51	0.0	0.13	Yt-2	0.37	0.0	0.13	Precision	8.82	0.0	0.28

USA (2020-2023)

Virus	Model	AIC	R²	pValueAC	Variable1	Coef1	pValue1	error1	Variable2	Coef2	pValue2	error2	Variable3	Coef3	pValue3	error3	Variable4	Coef4	pValue4	error4	Variable5	Coef5	pValue5	error5	Variable6	Coef6	pValue6	error6
IVA	None	-2077.0	0.0	0.0	Intercept	-6.71	0.0	0.32	Precision	5.33	0.0	0.36																
IVA	Yt-1	-2177.0	0.49	0.63	Intercept	-7.35	0.0	0.24	Yt-1	0.71	0.0	0.06	Precision	6.43	0.0	0.31												
IVA	Temp	-2082.0	0.04	0.0	Intercept	-6.73	0.0	0.31	Temp	-0.2	0.01	0.15	Precision	5.38	0.0	0.36												
IVA	AH	-2081.0	0.04	0.0	Intercept	-6.73	0.0	0.31	AH	-0.2	0.01	0.15	Precision	5.38	0.0	0.36												
IVA	Yt-1/2	-2176.0	0.5	0.03	Intercept	-7.35	0.0	0.24	Yt-1	0.65	0.0	0.19	Yt-2	0.07	0.49	0.2	Precision	6.44	0.0	0.31								
IVA	Temp_Yt-1	-2179.0	0.51	0.24	Intercept	-7.37	0.0	0.24	Temp	-0.14	0.06	0.14	Yt-1	0.68	0.0	0.07	Precision	6.47	0.0	0.31								
IVA	AH_Yt-1	-2180.0	0.51	0.25	Intercept	-7.37	0.0	0.24	AH	-0.17	0.02	0.15	Yt-1	0.68	0.0	0.07	Precision	6.48	0.0	0.31								
IVA	Temp_RH	-2080.0	0.05	0.0	Intercept	-6.73	0.0	0.31	Temp	-0.2	0.01	0.15	RH	0.03	0.74	0.16	Precision	5.38	0.0	0.36								
IVA	AH_RH	-2080.0	0.05	0.0	Intercept	-6.73	0.0	0.31	AH	-0.21	0.01	0.15	RH	0.07	0.36	0.16	Precision	5.38	0.0	0.36								
IVA	Temp_Yt-1/2	-2177.0	0.51	0.03	Intercept	-7.37	0.0	0.24	Temp	-0.13	0.07	0.15	Yt-1	0.65	0.0	0.18	Yt-2	0.04	0.7	0.19	Precision	6.47	0.0	0.31				
IVA	AH_Yt-1/2	-2179.0	0.51	0.02	Intercept	-7.38	0.0	0.24	AH	-0.16	0.03	0.15	Yt-1	0.64	0.0	0.18	Yt-2	0.05	0.62	0.19	Precision	6.49	0.0	0.31				
IVA	Temp_RH_Yt-1	-2179.0	0.51	0.19	Intercept	-7.38	0.0	0.24	Temp	-0.14	0.06	0.15	RH	-0.12	0.1	0.14	Yt-1	0.72	0.0	0.08	Precision	6.49	0.0	0.3				
IVA	AH_RH_Yt-1	-2180.0	0.52	0.25	Intercept	-7.38	0.0	0.24	AH	-0.15	0.05	0.15	RH	-0.09	0.23	0.14	Yt-1	0.71	0.0	0.08	Precision	6.5	0.0	0.3				
IVA	Temp_RH_Yt-1/2	-2178.0	0.52	0.01	Intercept	-7.39	0.0	0.24	Temp	-0.13	0.1	0.15	RH	-0.13	0.07	0.14	Yt-1	0.65	0.0	0.18	Yt-2	0.09	0.39	0.2	Precision	6.51	0.0	0.3
IVA	AH_RH_Yt-1/2	-2179.0	0.52	0.01	Intercept	-7.39	0.0	0.24	AH	-0.14	0.08	0.15	RH	-0.11	0.15	0.15	Yt-1	0.65	0.0	0.18	Yt-2	0.09	0.37	0.19	Precision	6.51	0.0	0.31
RSV	None	-1832.0	0.0	0.0	Intercept	-6.7	0.0	0.25	Precision	5.78	0.0	0.31																
RSV	Yt-1	-1952.0	0.56	0.0	Intercept	-7.1	0.0	0.18	Yt-1	0.65	0.0	0.06	Precision	6.78	0.0	0.27												
RSV	Temp	-1830.0	0.0	0.0	Intercept	-6.7	0.0	0.25	Temp	0.03	0.72	0.15	Precision	5.78	0.0	0.31												
RSV	AH	-1830.0	0.0	0.0	Intercept	-6.7	0.0	0.25	AH	0.04	0.56	0.15	Precision	5.79	0.0	0.31												
RSV	Yt-1/2	-1950.0	0.56	0.0	Intercept	-7.1	0.0	0.18	Yt-1	0.63	0.0	0.3	Yt-2	0.02	0.89	0.31	Precision	6.78	0.0	0.27								
RSV	Temp_Yt-1	-1956.0	0.57	0.0	Intercept	-7.12	0.0	0.18	Temp	0.16	0.02	0.14	Yt-1	0.68	0.0	0.07	Precision	6.83	0.0	0.27								
RSV	AH_Yt-1	-1956.0	0.57	0.0	Intercept	-7.12	0.0	0.18	AH	0.16	0.02	0.13	Yt-1	0.68	0.0	0.06	Precision	6.83	0.0	0.27								
RSV	Temp_RH	-1831.0	0.02	0.0	Intercept	-6.71	0.0	0.25	Temp	0.05	0.52	0.14	RH	0.14	0.09	0.16	Precision	5.81	0.0	0.31								
RSV	AH_RH	-1831.0	0.02	0.0	Intercept	-6.71	0.0	0.25	AH	0.04	0.61	0.14	RH	0.13	0.1	0.15	Precision	5.81	0.0	0.31								
RSV	Temp_Yt-1/2	-1954.0	0.57	0.0	Intercept	-7.12	0.0	0.18	Temp	0.18	0.01	0.14	Yt-1	0.57	0.0	0.29	Yt-2	0.12	0.42	0.3	Precision	6.83	0.0	0.27				
RSV	AH_Yt-1/2	-1954.0	0.57	0.0	Intercept	-7.12	0.0	0.18	AH	0.17	0.01	0.14	Yt-1	0.58	0.0	0.28	Yt-2	0.11	0.49	0.3	Precision	6.84	0.0	0.27				
RSV	Temp_RH_Yt-1	-1955.0	0.58	0.0	Intercept	-7.13	0.0	0.18	Temp	0.17	0.01	0.13	RH	0.08	0.22	0.13	Yt-1	0.68	0.0	0.07	Precision	6.85	0.0	0.27				
RSV	AH_RH_Yt-1	-1954.0	0.57	0.0	Intercept	-7.13	0.0	0.18	AH	0.15	0.02	0.13	RH	0.05	0.45	0.13	Yt-1	0.67	0.0	0.07	Precision	6.84	0.0	0.27				
RSV	Temp_RH_Yt-1/2	-1954.0	0.58	0.0	Intercept	-7.14	0.0	0.18	Temp	0.18	0.01	0.14	RH	0.08	0.24	0.13	Yt-1	0.58	0.0	0.27	Yt-2	0.11	0.47	0.29	Precision	6.86	0.0	0.27
RSV	AH_RH_Yt-1/2	-1953.0	0.58	0.0	Intercept	-7.13	0.0	0.18	AH	0.16	0.02	0.14	RH	0.05	0.5	0.13	Yt-1	0.59	0.0	0.28	Yt-2	0.09	0.55	0.29	Precision	6.84	0.0	0.27
hCoVs	None	-2016.0	0.0	0.0	Intercept	-7.66	0.0	0.2	Precision	7.24	0.0	0.28																
hCoVs	Yt-1	-2153.0	0.6	0.63	Intercept	-7.94	0.0	0.13	Yt-1	0.6	0.0	0.05	Precision	8.25	0.0	0.24												
hCoVs	Temp	-2022.0	0.05	0.0	Intercept	-7.68	0.0	0.19	Temp	-0.19	0.0	0.13	Precision	7.3	0.0	0.27												
hCoVs	AH	-2022.0	0.05	0.0	Intercept	-7.68	0.0	0.19	AH	-0.2	0.0	0.14	Precision	7.3	0.0	0.27												
hCoVs	Yt-1/2	-2152.0	0.61	0.03	Intercept	-7.95	0.0	0.14	Yt-1	0.53	0.0	0.18	Yt-2	0.08	0.42	0.2	Precision	8.26	0.0	0.24								
hCoVs	Temp_Yt-1	-2155.0	0.62	0.5	Intercept	-7.96	0.0	0.13	Temp	-0.12	0.04	0.11	Yt-1	0.58	0.0	0.06	Precision	8.3	0.0	0.25								
hCoVs	AH_Yt-1	-2157.0	0.62	0.48	Intercept	-7.97	0.0	0.13	AH	-0.15	0.01	0.12	Yt-1	0.58	0.0	0.06	Precision	8.32	0.0	0.25								
hCoVs	Temp_RH	-2021.0	0.06	0.0	Intercept	-7.68	0.0	0.19	Temp	-0.2	0.0	0.13	RH	-0.09	0.25	0.15	Precision	7.31	0.0	0.27								
hCoVs	AH_RH	-2021.0	0.06	0.0	Intercept	-7.68	0.0	0.19	AH	-0.2	0.0	0.14	RH	-0.04	0.57	0.15	Precision	7.31	0.0	0.27								
hCoVs	Temp_Yt-1/2	-2154.0	0.62	0.01	Intercept	-7.97	0.0	0.14	Temp	-0.12	0.04	0.11	Yt-1	0.52	0.0	0.18	Yt-2	0.07	0.44	0.19	Precision	8.3	0.0	0.25				
hCoVs	AH_Yt-1/2	-2156.0	0.62	0.02	Intercept	-7.97	0.0	0.13	AH	-0.15	0.01	0.12	Yt-1	0.53	0.0	0.17	Yt-2	0.06	0.53	0.19	Precision	8.32	0.0	0.25				
hCoVs	Temp_RH_Yt-1	-2167.0	0.65	0.87	Intercept	-8.0	0.0	0.13	Temp	-0.16	0.01	0.11	RH	-0.2	0.0	0.1	Yt-1	0.64	0.0	0.06	Precision	8.42	0.0	0.24				
hCoVs	AH_RH_Yt-1	-2165.0	0.65	0.8	Intercept	-8.0	0.0	0.13	AH	-0.14	0.02	0.12	RH	-0.17	0.0	0.1	Yt-1	0.63	0.0	0.06	Precision	8.4	0.0	0.25				
hCoVs	Temp_RH_Yt-1/2	-2165.0	0.65	0.37	Intercept	-8.0	0.0	0.13	Temp	-0.15	0.01	0.11	RH	-0.2	0.0	0.1	Yt-1	0.6	0.0	0.17	Yt-2	0.04	0.67	0.18	Precision	8.42	0.0	0.24
hCoVs	AH_RH_Yt-1/2	-2164.0	0.65	0.29	Intercept	-8.0	0.0	0.13	AH	-0.14	0.02	0.12	RH	-0.17	0.0	0.1	Yt-1	0.6	0.0	0.17	Yt-2	0.03	0.72	0.18	Precision	8.41	0.0	0.25
IVB	None	-2734.0	-0.0	0.85	Intercept	-9.99	0.0	0.21	Precision	9.42	0.0	0.29																
IVB	Yt-1	-2956.0	0.77	1.0	Intercept	-10.72	0.0	0.12	Yt-1	0.54	0.0	0.02	Precision	11.3	0.0	0.24												
IVB	Temp	-2736.0	0.03	0.85	Intercept	-10.0	0.0	0.21	Temp	-0.14	0.04	0.14	Precision	9.45	0.0	0.28												
IVB	AH	-2736.0	0.02	0.84	Intercept	-10.0	0.0	0.21	AH	-0.13	0.07	0.14	Precision	9.44	0.0	0.28												
IVB	Yt-1/2	-2967.0	0.79	0.72	Intercept	-10.75	0.0	0.12	Yt-1	0.04	0.67	0.2	Yt-2	0.53	0.0	0.21	Precision	11.41	0.0	0.24								
IVB	Temp_Yt-1	-2971.0	0.8	1.0	Intercept	-10.75	0.0	0.11	Temp	-0.22	0.0	0.1	Yt-1	0.53	0.0	0.02	Precision	11.44	0.0	0.24								
IVB	AH_Yt-1	-2965.0	0.79	1.0	Intercept	-10.73	0.0	0.12	AH	-0.18	0.0	0.1	Yt-1	0.53	0.0	0.02	Precision	11.39	0.0	0.24								
IVB	Temp_RH	-2737.0	0.04	0.86	Intercept	-10.01	0.0	0.21	Temp	-0.13	0.05	0.13	RH	0.12	0.09	0.14	Precision	9.47	0.0	0.28								
IVB	AH_RH	-2738.0	0.05	0.86	Intercept	-10.01	0.0	0.21	AH	-0.15	0.03	0.14	RH	0.15	0.03	0.14	Precision	9.48	0.0	0.28								
IVB	Temp_Yt-1/2	-2984.0	0.82	0.49	Intercept	-10.78	0.0	0.11	Temp	-0.22	0.0	0.1	Yt-1	0.06	0.56	0.19	Yt-2	0.51	0.0	0.2	Precision	11.56	0.0	0.24				
IVB	AH_Yt-1/2	-2978.0	0.81	0.65	Intercept	-10.76	0.0	0.11	AH	-0.18	0.0	0.1	Yt-1	0.05	0.59	0.19	Yt-2	0.51	0.0	0.2	Precision	11.5	0.0	0.24				
IVB	Temp_RH_Yt-1	-2991.0	0.83	0.01	Intercept	-10.8	0.0	0.11	Temp	-0.19	0.0	0.09	RH	0.2	0.0	0.08	Yt-1	0.53	0.0	0.02	Precision	11.62	0.0	0.24				
IVB	AH_RH_Yt-1	-2995.0	0.83	0.01	Intercept	-10.81	0.0	0.11	AH	-0.22	0.0	0.09	RH	0.24	0.0	0.08	Yt-1	0.53	0.0	0.02	Precision	11.66	0.0	0.24				
IVB	Temp_RH_Yt-1/2	-3007.0	0.85	0.05	Intercept	-10.84	0.0	0.11	Temp	-0.2	0.0	0.09	RH	0.21	0.0	0.08	Yt-1	0.1	0.2	0.16	Yt-2	0.46	0.0	0.17	Precision	11.77	0.0	0.24
IVB	AH_RH_Yt-1/2	-3011.0	0.85	0.07	Intercept	-10.85	0.0	0.1	AH	-0.23	0.0	0.09	RH	0.25	0.0	0.07	Yt-1	0.11	0.16	0.16	Yt-2	0.44	0.0	0.17	Precision	11.8	0.0	0.24
hMPV	None	-2094.0	0.0	0.0	Intercept	-7.5	0.0	0.26	Precision	6.54	0.0	0.32																
hMPV	Yt-1	-2203.0	0.52	0.04	Intercept	-7.85	0.0	0.19	Yt-1	0.62	0.0	0.06	Precision	7.41	0.0	0.27												
hMPV	Temp	-2096.0	0.03	0.0	Intercept	-7.52	0.0	0.26	Temp	-0.14	0.05	0.14	Precision	6.56	0.0	0.31												
hMPV	AH	-2095.0	0.02	0.0	Intercept	-7.51	0.0	0.26	AH	-0.13	0.07	0.15	Precision	6.56	0.0	0.32												
hMPV	Yt-1/2	-2201.0	0.52	0.01	Intercept	-7.85	0.0	0.19	Yt-1	0.6	0.0	0.22	Yt-2	0.02	0.86	0.24	Precision	7.41	0.0	0.27								
hMPV	Temp_Yt-1	-2202.0	0.53	0.03	Intercept	-7.87	0.0	0.19	Temp	-0.09	0.22	0.14	Yt-1	0.61	0.0	0.06	Precision	7.44	0.0	0.27								
hMPV	AH_Yt-1	-2202.0	0.53	0.02	Intercept	-7.87	0.0	0.19	AH	-0.09	0.21	0.14	Yt-1	0.62	0.0	0.06	Precision	7.44	0.0	0.27								
hMPV	Temp_RH	-2095.0	0.03	0.0	Intercept	-7.52	0.0	0.26	Temp	-0.13	0.06	0.14	RH	0.07	0.34	0.15	Precision	6.57	0.0	0.32								
hMPV	AH_RH	-2095.0	0.03	0.0	Intercept	-7.52	0.0	0.26	AH	-0.14	0.05	0.14	RH	0.11	0.17	0.15	Precision	6.57	0.0	0.31								
hMPV	Temp_Yt-1/2	-2200.0	0.53	0.01	Intercept	-7.87	0.0	0.19	Temp	-0.09	0.22	0.14	Yt-1	0.6	0.0	0.22	Yt-2	0.01	0.92	0.24	Precision	7.44	0.0	0.27				
hMPV	AH_Yt-1/2	-2200.0	0.53	0.01	Intercept	-7.87	0.0	0.19	AH	-0.09	0.22	0.14	Yt-1	0.61	0.0	0.22	Yt-2	0.0	0.98	0.24	Precision	7.44	0.0	0.27				
hMPV	Temp_RH_Yt-1	-2200.0	0.53	0.03	Intercept	-7.87	0.0	0.19	Temp	-0.09	0.2	0.14	RH	-0.03	0.65	0.14	Yt-1	0.62	0.0	0.07	Precision	7.44	0.0	0.27				
hMPV	AH_RH_Yt-1	-2200.0	0.53	0.02	Intercept	-7.87	0.0	0.19	AH	-0.09	0.22	0.14	RH	-0.01	0.87	0.14	Yt-1	0.62	0.0	0.07	Precision	7.44	0.0	0.27				
hMPV	Temp_RH_Yt-1/2	-2198.0	0.53	0.01	Intercept	-7.87	0.0	0.19	Temp	-0.09	0.2	0.14	RH	-0.03	0.66	0.14	Yt-1	0.62	0.0	0.24	Yt-2	-0.0	0.98	0.25	Precision	7.44	0.0	0.27
hMPV	AH_RH_Yt-1/2	-2198.0	0.53	0.01	Intercept	-7.87	0.0	0.19	AH	-0.09	0.22	0.14	RH	-0.01	0.87	0.14	Yt-1	0.62	0.0	0.24	Yt-2	-0.0	0.99	0.25	Precision	7.44	0.0	0.27
