# Supplementary material for: The effects of weather and mobility on respiratory viruses dynamics before and during the COVID-19 pandemic in the USA and Canada
Source: PLOS Digit Health. 2023 Dec 21;2(12):e0000405. doi: 10.1371/journal.pdig.0000405 (PMC10734953; doi:10.1371/journal.pdig.0000405)
Supplement: S3 Table — (PDF) [file pdig.0000405.s016.pdf]

S3 Table. Regression models results for the number of trips analysis for the USA.

pValueAC, p-value autocorrelation (AC) Breusch-Godfrey test up to 5 (pre-pandemic) or 53 weeks (pandemic); Coef, regression coefficient; Temp, temperature; AH, absolute humidity; RH, relative humidity; Yt-1, AC term 1 week; N_Trips, number of trips. Models used for comparison in grey.

Pre-COVID-19 pandemic (January 2019-March 2020)

Virus	Model	AIC	R²	pValueAC	Variable1	Coef1	pValue1	error1	Variable2	Coef2	pValue2	error2	Variable3	Coef3	pValue3	error3	Variable4	Coef4	pValue4	error4	Variable5	Coef5	pValue5	error5	Variable6	Coef6	pValue6	error6
IVA	Yt-1	-688.0	0.79	0.0	Intercept	-6.17	0.0	0.2	Yt-1	1.02	0.0	0.12	Precision	6.65	0.0	0.38												
IVA	Temp_Yt-1	-727.1	0.89	0.01	Intercept	-6.39	0.0	0.17	Temp	-0.77	0.0	0.19	Yt-1	0.69	0.0	0.11	Precision	7.46	0.0	0.37								
IVA	AH_Yt-1	-722.9	0.88	0.0	Intercept	-6.41	0.0	0.18	AH	-0.79	0.0	0.23	Yt-1	0.73	0.0	0.11	Precision	7.44	0.0	0.38								
IVA	NTrips_Yt-1	-702.7	0.84	0.0	Intercept	-6.25	0.0	0.19	N_Trips	-0.5	0.0	0.22	Yt-1	0.72	0.0	0.17	Precision	6.96	0.0	0.37								
IVA	AH_RH_Yt-1	-728.6	0.9	0.0	Intercept	-6.44	0.0	0.18	AH	-0.82	0.0	0.22	RH	0.12	0.0	0.08	Yt-1	0.72	0.0	0.1	Precision	7.58	0.0	0.38				
IVA	Temp_RH_Yt-1	-725.5	0.89	0.01	Intercept	-6.38	0.0	0.17	Temp	-0.76	0.0	0.2	RH	0.03	0.55	0.1	Yt-1	0.7	0.0	0.11	Precision	7.46	0.0	0.37				
IVA	Temp_Yt-1_NTrips	-725.1	0.89	0.01	Intercept	-6.39	0.0	0.17	Temp	-0.77	0.0	0.27	N_Trips	0.0	0.97	0.23	Yt-1	0.7	0.0	0.12	Precision	7.46	0.0	0.37				
IVA	AH_Yt-1_NTrips	-725.1	0.89	0.0	Intercept	-6.42	0.0	0.18	AH	-0.69	0.0	0.25	N_Trips	-0.19	0.04	0.18	Yt-1	0.67	0.0	0.12	Precision	7.51	0.0	0.38				
IVA	Temp_RH_Yt-1_NTrips	-723.6	0.89	0.01	Intercept	-6.38	0.0	0.17	Temp	-0.8	0.0	0.27	RH	0.04	0.46	0.11	N_Trips	0.06	0.67	0.27	Yt-1	0.71	0.0	0.13	Precision	7.47	0.0	0.37
IVA	AH_RH_Yt-1_NTrips	-726.6	0.9	0.0	Intercept	-6.44	0.0	0.18	AH	-0.8	0.0	0.27	RH	0.11	0.05	0.11	N_Trips	-0.03	0.78	0.23	Yt-1	0.71	0.0	0.12	Precision	7.58	0.0	0.38
RSV	Yt-1	-436.4	0.74	0.08	Intercept	-5.78	0.0	0.16	Yt-1	0.61	0.0	0.1	Precision	7.13	0.0	0.44												
RSV	Temp_Yt-1	-472.9	0.9	0.42	Intercept	-5.9	0.0	0.11	Temp	-0.51	0.0	0.13	Yt-1	0.47	0.0	0.09	Precision	8.12	0.0	0.43								
RSV	AH_Yt-1	-465.9	0.88	0.83	Intercept	-5.89	0.0	0.12	AH	-0.49	0.0	0.16	Yt-1	0.49	0.0	0.09	Precision	7.95	0.0	0.43								
RSV	NTrips_Yt-1	-443.6	0.79	0.03	Intercept	-5.82	0.0	0.14	N_Trips	-0.31	0.0	0.19	Yt-1	0.45	0.0	0.14	Precision	7.38	0.0	0.44								
RSV	AH_RH_Yt-1	-488.8	0.93	0.47	Intercept	-5.95	0.0	0.1	AH	-0.61	0.0	0.13	RH	0.23	0.0	0.08	Yt-1	0.39	0.0	0.08	Precision	8.58	0.0	0.43				
RSV	Temp_RH_Yt-1	-478.1	0.91	0.25	Intercept	-5.92	0.0	0.1	Temp	-0.51	0.0	0.12	RH	0.13	0.0	0.09	Yt-1	0.42	0.0	0.09	Precision	8.3	0.0	0.43				
RSV	Temp_Yt-1_NTrips	-474.4	0.9	0.64	Intercept	-5.91	0.0	0.11	Temp	-0.62	0.0	0.17	N_Trips	0.16	0.05	0.16	Yt-1	0.52	0.0	0.1	Precision	8.2	0.0	0.43				
RSV	AH_Yt-1_NTrips	-464.0	0.88	0.79	Intercept	-5.89	0.0	0.12	AH	-0.48	0.0	0.18	N_Trips	-0.02	0.82	0.17	Yt-1	0.48	0.0	0.11	Precision	7.95	0.0	0.43				
RSV	Temp_RH_Yt-1_NTrips	-484.0	0.93	0.8	Intercept	-5.94	0.0	0.1	Temp	-0.66	0.0	0.14	RH	0.15	0.0	0.08	N_Trips	0.22	0.0	0.14	Yt-1	0.48	0.0	0.09	Precision	8.5	0.0	0.43
RSV	AH_RH_Yt-1_NTrips	-492.5	0.94	0.63	Intercept	-5.96	0.0	0.09	AH	-0.72	0.0	0.15	RH	0.27	0.0	0.08	N_Trips	0.15	0.01	0.12	Yt-1	0.43	0.0	0.08	Precision	8.72	0.0	0.43
hCoVs	Yt-1	-800.2	0.83	0.01	Intercept	-7.04	0.0	0.17	Yt-1	1.04	0.0	0.11	Precision	7.96	0.0	0.37												
hCoVs	Temp_Yt-1	-837.6	0.91	0.31	Intercept	-7.2	0.0	0.14	Temp	-0.7	0.0	0.18	Yt-1	0.65	0.0	0.13	Precision	8.69	0.0	0.36								
hCoVs	AH_Yt-1	-839.3	0.91	0.43	Intercept	-7.23	0.0	0.14	AH	-0.73	0.0	0.19	Yt-1	0.71	0.0	0.11	Precision	8.76	0.0	0.37								
hCoVs	NTrips_Yt-1	-803.6	0.85	0.0	Intercept	-7.07	0.0	0.16	N_Trips	-0.35	0.02	0.29	Yt-1	0.75	0.0	0.26	Precision	8.06	0.0	0.37								
hCoVs	AH_RH_Yt-1	-846.8	0.93	0.33	Intercept	-7.27	0.0	0.14	AH	-0.8	0.0	0.18	RH	0.12	0.0	0.07	Yt-1	0.65	0.0	0.1	Precision	8.94	0.0	0.37				
hCoVs	Temp_RH_Yt-1	-837.4	0.91	0.27	Intercept	-7.2	0.0	0.14	Temp	-0.7	0.0	0.17	RH	0.06	0.18	0.08	Yt-1	0.63	0.0	0.13	Precision	8.72	0.0	0.36				
hCoVs	Temp_Yt-1_NTrips	-838.7	0.92	0.41	Intercept	-7.21	0.0	0.14	Temp	-0.82	0.0	0.22	N_Trips	0.21	0.06	0.22	Yt-1	0.75	0.0	0.17	Precision	8.75	0.0	0.36				
hCoVs	AH_Yt-1_NTrips	-837.3	0.91	0.42	Intercept	-7.23	0.0	0.14	AH	-0.73	0.0	0.21	N_Trips	0.01	0.95	0.19	Yt-1	0.72	0.0	0.16	Precision	8.76	0.0	0.37				
hCoVs	Temp_RH_Yt-1_NTrips	-842.9	0.92	0.75	Intercept	-7.23	0.0	0.13	Temp	-0.92	0.0	0.21	RH	0.11	0.01	0.08	N_Trips	0.37	0.0	0.25	Yt-1	0.79	0.0	0.16	Precision	8.86	0.0	0.36
hCoVs	AH_RH_Yt-1_NTrips	-850.1	0.93	0.52	Intercept	-7.3	0.0	0.14	AH	-0.95	0.0	0.21	RH	0.18	0.0	0.08	N_Trips	0.25	0.02	0.2	Yt-1	0.77	0.0	0.14	Precision	9.05	0.0	0.37
hMPV	Yt-1	-546.9	0.85	0.02	Intercept	-6.57	0.0	0.08	Yt-1	0.55	0.0	0.07	Precision	9.34	0.0	0.43												
hMPV	Temp_Yt-1	-553.6	0.88	0.23	Intercept	-6.58	0.0	0.07	Temp	-0.14	0.0	0.09	Yt-1	0.5	0.0	0.07	Precision	9.55	0.0	0.43								
hMPV	AH_Yt-1	-551.2	0.87	0.31	Intercept	-6.58	0.0	0.07	AH	-0.13	0.01	0.1	Yt-1	0.5	0.0	0.07	Precision	9.49	0.0	0.43								
hMPV	NTrips_Yt-1	-548.8	0.87	0.08	Intercept	-6.57	0.0	0.08	N_Trips	-0.09	0.04	0.09	Yt-1	0.5	0.0	0.08	Precision	9.43	0.0	0.43								
hMPV	AH_RH_Yt-1	-554.7	0.89	0.17	Intercept	-6.58	0.0	0.07	AH	-0.12	0.01	0.1	RH	0.07	0.02	0.06	Yt-1	0.51	0.0	0.07	Precision	9.62	0.0	0.43				
hMPV	Temp_RH_Yt-1	-553.7	0.89	0.13	Intercept	-6.58	0.0	0.07	Temp	-0.11	0.02	0.09	RH	0.05	0.14	0.07	Yt-1	0.51	0.0	0.07	Precision	9.6	0.0	0.43				
hMPV	Temp_Yt-1_NTrips	-551.8	0.88	0.28	Intercept	-6.58	0.0	0.07	Temp	-0.16	0.02	0.14	N_Trips	0.03	0.64	0.13	Yt-1	0.5	0.0	0.07	Precision	9.55	0.0	0.43				
hMPV	AH_Yt-1_NTrips	-549.8	0.88	0.25	Intercept	-6.58	0.0	0.07	AH	-0.1	0.09	0.12	N_Trips	-0.04	0.46	0.1	Yt-1	0.49	0.0	0.08	Precision	9.5	0.0	0.43				
hMPV	Temp_RH_Yt-1_NTrips	-554.1	0.89	0.19	Intercept	-6.58	0.0	0.07	Temp	-0.2	0.0	0.14	RH	0.08	0.03	0.08	N_Trips	0.12	0.11	0.14	Yt-1	0.55	0.0	0.08	Precision	9.66	0.0	0.43
hMPV	AH_RH_Yt-1_NTrips	-554.4	0.89	0.26	Intercept	-6.58	0.0	0.07	AH	-0.18	0.0	0.13	RH	0.11	0.01	0.08	N_Trips	0.09	0.18	0.13	Yt-1	0.54	0.0	0.08	Precision	9.67	0.0	0.43

Pandemic (March 2020-March 2022)

Virus	Model	AIC	R^2	pValueAC	Variable1	Coef1	pValue1	error1	Variable2	Coef2	pValue2	error2	Variable3	Coef3	pValue3	error3	Variable4	Coef4	pValue4	error4	Variable5	Coef5	pValue5	error5	Variable6	Coef6	pValue6	error6
IVA	Yt-1	-1771.0	0.42	0.01	Intercept	-8.67	0.0	0.29	Yt-1	0.57	0.0	0.06	Precision	7.77	0.0	0.37												
IVA	Temp_Yt-1	-1778.0	0.47	0.0	Intercept	-8.73	0.0	0.28	Temp	-0.27	0.0	0.18	Yt-1	0.55	0.0	0.06	Precision	7.89	0.0	0.36								
IVA	AH_Yt-1	-1776.0	0.46	0.01	Intercept	-8.71	0.0	0.29	AH	-0.25	0.01	0.18	Yt-1	0.55	0.0	0.06	Precision	7.86	0.0	0.36								
IVA	NTrips_Yt-1	-1770.0	0.43	0.05	Intercept	-8.68	0.0	0.29	N_Trips	0.12	0.21	0.18	Yt-1	0.57	0.0	0.06	Precision	7.79	0.0	0.36								
IVA	AH_RH_Yt-1	-1775.0	0.46	0.0	Intercept	-8.72	0.0	0.29	AH	-0.26	0.0	0.18	RH	0.07	0.41	0.16	Yt-1	0.55	0.0	0.06	Precision	7.88	0.0	0.36				
IVA	Temp_RH_Yt-1	-1776.0	0.47	0.0	Intercept	-8.73	0.0	0.28	Temp	-0.27	0.0	0.18	RH	0.01	0.92	0.16	Yt-1	0.55	0.0	0.06	Precision	7.89	0.0	0.36				
IVA	Temp_Yt-1_NTrips	-1780.0	0.49	0.06	Intercept	-8.77	0.0	0.28	Temp	-0.32	0.0	0.18	N_Trips	0.2	0.04	0.19	Yt-1	0.55	0.0	0.06	Precision	7.97	0.0	0.36				
IVA	AH_Yt-1_NTrips	-1777.0	0.47	0.09	Intercept	-8.73	0.0	0.28	AH	-0.28	0.0	0.19	N_Trips	0.16	0.09	0.18	Yt-1	0.55	0.0	0.06	Precision	7.91	0.0	0.36				
IVA	Temp_RH_Yt-1_NTrips	-1779.0	0.49	0.02	Intercept	-8.79	0.0	0.29	Temp	-0.33	0.0	0.18	RH	0.08	0.33	0.17	N_Trips	0.23	0.02	0.2	Yt-1	0.55	0.0	0.06	Precision	8.0	0.0	0.37
IVA	AH_RH_Yt-1_NTrips	-1778.0	0.49	0.02	Intercept	-8.79	0.0	0.29	AH	-0.32	0.0	0.19	RH	0.15	0.08	0.17	N_Trips	0.23	0.02	0.2	Yt-1	0.55	0.0	0.06	Precision	7.99	0.0	0.37
RSV	Yt-1	-1578.0	0.77	0.03	Intercept	-7.94	0.0	0.17	Yt-1	1.07	0.0	0.09	Precision	8.11	0.0	0.3												
RSV	Temp_Yt-1	-1579.0	0.78	0.06	Intercept	-7.97	0.0	0.18	Temp	-0.11	0.07	0.12	Yt-1	1.12	0.0	0.1	Precision	8.16	0.0	0.3								
RSV	AH_Yt-1	-1580.0	0.78	0.07	Intercept	-7.97	0.0	0.17	AH	-0.12	0.05	0.12	Yt-1	1.13	0.0	0.11	Precision	8.17	0.0	0.3								
RSV	NTrips_Yt-1	-1628.0	0.86	0.38	Intercept	-8.14	0.0	0.16	N_Trips	0.61	0.0	0.16	Yt-1	0.93	0.0	0.08	Precision	8.73	0.0	0.29								
RSV	AH_RH_Yt-1	-1578.0	0.78	0.07	Intercept	-7.97	0.0	0.18	AH	-0.12	0.05	0.12	RH	-0.02	0.72	0.14	Yt-1	1.13	0.0	0.11	Precision	8.16	0.0	0.3				
RSV	Temp_RH_Yt-1	-1578.0	0.78	0.06	Intercept	-7.96	0.0	0.18	Temp	-0.12	0.06	0.13	RH	-0.05	0.49	0.14	Yt-1	1.13	0.0	0.11	Precision	8.16	0.0	0.3				
RSV	Temp_Yt-1_NTrips	-1626.0	0.86	0.39	Intercept	-8.15	0.0	0.16	Temp	-0.04	0.43	0.11	N_Trips	0.59	0.0	0.16	Yt-1	0.96	0.0	0.11	Precision	8.75	0.0	0.3				
RSV	AH_Yt-1_NTrips	-1626.0	0.86	0.4	Intercept	-8.15	0.0	0.16	AH	-0.04	0.51	0.11	N_Trips	0.59	0.0	0.17	Yt-1	0.96	0.0	0.11	Precision	8.74	0.0	0.29				
RSV	Temp_RH_Yt-1_NTrips	-1628.0	0.87	0.16	Intercept	-8.17	0.0	0.15	Temp	-0.02	0.72	0.11	RH	0.11	0.04	0.1	N_Trips	0.64	0.0	0.17	Yt-1	0.91	0.0	0.12	Precision	8.8	0.0	0.29
RSV	AH_RH_Yt-1_NTrips	-1629.0	0.87	0.18	Intercept	-8.18	0.0	0.15	AH	-0.04	0.53	0.11	RH	0.11	0.03	0.1	N_Trips	0.63	0.0	0.17	Yt-1	0.92	0.0	0.12	Precision	8.81	0.0	0.29
hCoVs	Yt-1	-1558.0	0.52	0.99	Intercept	-8.17	0.0	0.18	Yt-1	0.51	0.0	0.06	Precision	8.28	0.0	0.3												
hCoVs	Temp_Yt-1	-1558.0	0.52	0.99	Intercept	-8.18	0.0	0.18	Temp	-0.1	0.2	0.15	Yt-1	0.5	0.0	0.06	Precision	8.3	0.0	0.3								
hCoVs	AH_Yt-1	-1558.0	0.53	0.99	Intercept	-8.18	0.0	0.18	AH	-0.11	0.14	0.15	Yt-1	0.5	0.0	0.06	Precision	8.31	0.0	0.3								
hCoVs	NTrips_Yt-1	-1687.0	0.86	0.96	Intercept	-8.53	0.0	0.11	N_Trips	0.73	0.0	0.1	Yt-1	0.67	0.0	0.04	Precision	9.72	0.0	0.28								
hCoVs	AH_RH_Yt-1	-1562.0	0.55	1.0	Intercept	-8.2	0.0	0.17	AH	-0.1	0.22	0.16	RH	-0.18	0.02	0.15	Yt-1	0.54	0.0	0.07	Precision	8.38	0.0	0.3				
hCoVs	Temp_RH_Yt-1	-1563.0	0.55	1.0	Intercept	-8.21	0.0	0.17	Temp	-0.12	0.11	0.15	RH	-0.2	0.01	0.14	Yt-1	0.54	0.0	0.07	Precision	8.39	0.0	0.3				
hCoVs	Temp_Yt-1_NTrips	-1685.0	0.86	0.91	Intercept	-8.53	0.0	0.11	Temp	-0.02	0.7	0.08	N_Trips	0.72	0.0	0.1	Yt-1	0.67	0.0	0.05	Precision	9.72	0.0	0.28				
hCoVs	AH_Yt-1_NTrips	-1685.0	0.86	0.93	Intercept	-8.52	0.0	0.12	AH	-0.03	0.52	0.09	N_Trips	0.72	0.0	0.1	Yt-1	0.67	0.0	0.05	Precision	9.72	0.0	0.28				
hCoVs	Temp_RH_Yt-1_NTrips	-1685.0	0.86	0.97	Intercept	-8.52	0.0	0.11	Temp	-0.02	0.71	0.08	RH	-0.06	0.13	0.08	N_Trips	0.71	0.0	0.1	Yt-1	0.68	0.0	0.05	Precision	9.74	0.0	0.28
hCoVs	AH_RH_Yt-1_NTrips	-1685.0	0.86	0.97	Intercept	-8.52	0.0	0.11	AH	-0.01	0.75	0.09	RH	-0.06	0.16	0.08	N_Trips	0.72	0.0	0.1	Yt-1	0.68	0.0	0.05	Precision	9.74	0.0	0.28
hMPV	Yt-1	-1678.0	0.53	0.76	Intercept	-8.23	0.0	0.25	Yt-1	0.72	0.0	0.08	Precision	7.56	0.0	0.34												
hMPV	Temp_Yt-1	-1678.0	0.54	0.77	Intercept	-8.27	0.0	0.26	Temp	-0.13	0.14	0.17	Yt-1	0.71	0.0	0.08	Precision	7.61	0.0	0.34								
hMPV	AH_Yt-1	-1678.0	0.53	0.75	Intercept	-8.26	0.0	0.26	AH	-0.12	0.2	0.18	Yt-1	0.71	0.0	0.08	Precision	7.6	0.0	0.34								
hMPV	NTrips_Yt-1	-1719.0	0.68	0.97	Intercept	-8.56	0.0	0.23	N_Trips	0.51	0.0	0.14	Yt-1	0.88	0.0	0.08	Precision	8.21	0.0	0.33								
hMPV	AH_RH_Yt-1	-1676.0	0.53	0.73	Intercept	-8.26	0.0	0.26	AH	-0.12	0.19	0.18	RH	0.04	0.66	0.16	Yt-1	0.7	0.0	0.09	Precision	7.6	0.0	0.34				
hMPV	Temp_RH_Yt-1	-1676.0	0.54	0.76	Intercept	-8.27	0.0	0.26	Temp	-0.13	0.14	0.17	RH	0.01	0.92	0.16	Yt-1	0.71	0.0	0.09	Precision	7.61	0.0	0.34				
hMPV	Temp_Yt-1_NTrips	-1717.0	0.68	0.99	Intercept	-8.56	0.0	0.23	Temp	-0.05	0.51	0.16	N_Trips	0.5	0.0	0.14	Yt-1	0.86	0.0	0.09	Precision	8.21	0.0	0.33				
hMPV	AH_Yt-1_NTrips	-1717.0	0.68	0.98	Intercept	-8.56	0.0	0.23	AH	-0.05	0.56	0.16	N_Trips	0.5	0.0	0.14	Yt-1	0.87	0.0	0.08	Precision	8.21	0.0	0.33				
hMPV	Temp_RH_Yt-1_NTrips	-1716.0	0.69	0.91	Intercept	-8.56	0.0	0.23	Temp	-0.06	0.48	0.15	RH	0.07	0.26	0.13	N_Trips	0.5	0.0	0.14	Yt-1	0.84	0.0	0.1	Precision	8.21	0.0	0.33
hMPV	AH_RH_Yt-1_NTrips	-1717.0	0.69	0.9	Intercept	-8.56	0.0	0.23	AH	-0.08	0.36	0.16	RH	0.09	0.19	0.13	N_Trips	0.5	0.0	0.14	Yt-1	0.83	0.0	0.1	Precision	8.22	0.0	0.33
