# Supplementary material for: The effects of weather and mobility on respiratory viruses dynamics before and during the COVID-19 pandemic in the USA and Canada
Source: PLOS Digit Health. 2023 Dec 21;2(12):e0000405. doi: 10.1371/journal.pdig.0000405 (PMC10734953; doi:10.1371/journal.pdig.0000405)
Supplement: S5 Table — (PDF) [file pdig.0000405.s018.pdf]

S5 Table. Regression models results for the visits to transit stations analysis for Canada and the USA in the pandemic period (March 2020-October 2022).

pValueAC, p-value autocorrelation (AC) Breusch-Godfrey test up to 53 weeks; Coef, regression coefficient; Temp, temperature; AH, absolute humidity; RH, relative humidity; Yt-1, AC term 1 week; Transit, visit to transit stations. Models used for comparison in grey.

Canada (March 2020-October 2022)

Virus	Model	AIC	R^2	pValueAC	Variable1	Coef1	pValue1	error1	Variable2	Coef2	pValue2	error2	Variable3	Coef3	pValue3	error3	Variable4	Coef4	pValue4	error4	Variable5	Coef5	pValue5	error5	Variable6	Coef6	pValue6	error6
IVA	Yt-1	-2924.0	0.43	0.91	Intercept	-9.72	0.0	0.32	Yt-1	0.77	0.0	0.09	Precision	8.33	0.0	0.37												
IVA	Temp_Yt-1	-2922.0	0.43	0.88	Intercept	-9.72	0.0	0.32	Temp	-0.02	0.81	0.15	Yt-1	0.77	0.0	0.09	Precision	8.33	0.0	0.37								
IVA	AH_Yt-1	-2922.0	0.43	0.85	Intercept	-9.72	0.0	0.32	AH	-0.03	0.7	0.15	Yt-1	0.76	0.0	0.09	Precision	8.33	0.0	0.37								
IVA	Transit_Yt-1	-2936.0	0.49	0.71	Intercept	-9.77	0.0	0.3	Transit	0.31	0.0	0.15	Yt-1	0.68	0.0	0.1	Precision	8.45	0.0	0.36								
IVA	AH_RH_Yt-1	-2921.0	0.44	0.85	Intercept	-9.71	0.0	0.32	AH	-0.03	0.69	0.15	RH	0.02	0.74	0.14	Yt-1	0.77	0.0	0.09	Precision	8.32	0.0	0.37				
IVA	Temp_RH_Yt-1	-2920.0	0.43	0.88	Intercept	-9.71	0.0	0.32	Temp	-0.02	0.84	0.15	RH	0.02	0.77	0.14	Yt-1	0.77	0.0	0.09	Precision	8.32	0.0	0.37				
IVA	Temp_Yt-1_Transit	-2936.0	0.5	0.58	Intercept	-9.74	0.0	0.31	Temp	-0.11	0.15	0.16	Transit	0.34	0.0	0.16	Yt-1	0.65	0.0	0.11	Precision	8.42	0.0	0.36				
IVA	AH_Yt-1_Transit	-2939.0	0.51	0.57	Intercept	-9.73	0.0	0.3	AH	-0.17	0.04	0.17	Transit	0.37	0.0	0.16	Yt-1	0.63	0.0	0.12	Precision	8.43	0.0	0.36				
IVA	Temp_RH_Yt-1_Transit	-2936.0	0.5	0.53	Intercept	-9.76	0.0	0.31	Temp	-0.14	0.09	0.16	RH	-0.1	0.21	0.16	Transit	0.38	0.0	0.17	Yt-1	0.62	0.0	0.13	Precision	8.45	0.0	0.37
IVA	AH_RH_Yt-1_Transit	-2938.0	0.51	0.56	Intercept	-9.76	0.0	0.3	AH	-0.18	0.03	0.17	RH	-0.09	0.26	0.16	Transit	0.4	0.0	0.18	Yt-1	0.6	0.0	0.13	Precision	8.46	0.0	0.36
RSV	Yt-1	-2338.0	0.56	0.16	Intercept	-8.87	0.0	0.21	Yt-1	0.74	0.0	0.08	Precision	8.3	0.0	0.29												
RSV	Temp_Yt-1	-2340.0	0.57	0.12	Intercept	-8.88	0.0	0.21	Temp	0.14	0.07	0.15	Yt-1	0.79	0.0	0.1	Precision	8.34	0.0	0.29								
RSV	AH_Yt-1	-2337.0	0.56	0.16	Intercept	-8.87	0.0	0.21	AH	0.08	0.29	0.15	Yt-1	0.76	0.0	0.09	Precision	8.31	0.0	0.29								
RSV	Transit_Yt-1	-2367.0	0.65	0.66	Intercept	-9.0	0.0	0.19	Transit	0.36	0.0	0.11	Yt-1	0.77	0.0	0.08	Precision	8.61	0.0	0.28								
RSV	AH_RH_Yt-1	-2336.0	0.56	0.13	Intercept	-8.87	0.0	0.21	AH	0.08	0.27	0.15	RH	-0.06	0.43	0.15	Yt-1	0.78	0.0	0.1	Precision	8.32	0.0	0.29				
RSV	Temp_RH_Yt-1	-2338.0	0.57	0.1	Intercept	-8.88	0.0	0.21	Temp	0.14	0.08	0.15	RH	-0.04	0.56	0.15	Yt-1	0.81	0.0	0.11	Precision	8.34	0.0	0.29				
RSV	Temp_Yt-1_Transit	-2366.0	0.65	0.43	Intercept	-8.99	0.0	0.19	Temp	-0.08	0.29	0.16	Transit	0.38	0.0	0.11	Yt-1	0.74	0.0	0.11	Precision	8.61	0.0	0.28				
RSV	AH_Yt-1_Transit	-2368.0	0.65	0.45	Intercept	-8.99	0.0	0.19	AH	-0.13	0.1	0.15	Transit	0.39	0.0	0.11	Yt-1	0.73	0.0	0.1	Precision	8.62	0.0	0.28				
RSV	Temp_RH_Yt-1_Transit	-2364.0	0.65	0.24	Intercept	-8.99	0.0	0.19	Temp	-0.08	0.3	0.16	RH	-0.02	0.82	0.15	Transit	0.38	0.0	0.12	Yt-1	0.74	0.0	0.11	Precision	8.61	0.0	0.28
RSV	AH_RH_Yt-1_Transit	-2366.0	0.65	0.39	Intercept	-8.99	0.0	0.19	AH	-0.13	0.1	0.16	RH	0.0	0.99	0.14	Transit	0.39	0.0	0.11	Yt-1	0.73	0.0	0.1	Precision	8.62	0.0	0.28
hCoVs	Yt-1	-2311.0	0.47	0.96	Intercept	-8.94	0.0	0.22	Yt-1	0.58	0.0	0.07	Precision	8.35	0.0	0.29												
hCoVs	Temp_Yt-1	-2309.0	0.47	0.33	Intercept	-8.94	0.0	0.22	Temp	-0.02	0.77	0.16	Yt-1	0.58	0.0	0.09	Precision	8.35	0.0	0.29								
hCoVs	AH_Yt-1	-2310.0	0.47	0.51	Intercept	-8.95	0.0	0.22	AH	-0.08	0.32	0.16	Yt-1	0.56	0.0	0.08	Precision	8.37	0.0	0.29								
hCoVs	Transit_Yt-1	-2330.0	0.54	0.97	Intercept	-9.05	0.0	0.21	Transit	0.37	0.0	0.14	Yt-1	0.66	0.0	0.07	Precision	8.58	0.0	0.29								
hCoVs	AH_RH_Yt-1	-2311.0	0.49	0.52	Intercept	-8.97	0.0	0.21	AH	-0.09	0.28	0.16	RH	-0.12	0.07	0.13	Yt-1	0.57	0.0	0.08	Precision	8.4	0.0	0.29				
hCoVs	Temp_RH_Yt-1	-2311.0	0.48	0.46	Intercept	-8.96	0.0	0.21	Temp	-0.06	0.5	0.16	RH	-0.13	0.06	0.14	Yt-1	0.58	0.0	0.08	Precision	8.39	0.0	0.29				
hCoVs	Temp_Yt-1_Transit	-2345.0	0.6	0.65	Intercept	-9.13	0.0	0.2	Temp	-0.36	0.0	0.16	Transit	0.53	0.0	0.12	Yt-1	0.56	0.0	0.08	Precision	8.76	0.0	0.28				
hCoVs	AH_Yt-1_Transit	-2353.0	0.62	0.93	Intercept	-9.16	0.0	0.19	AH	-0.43	0.0	0.16	Transit	0.54	0.0	0.11	Yt-1	0.58	0.0	0.07	Precision	8.84	0.0	0.28				
hCoVs	Temp_RH_Yt-1_Transit	-2351.0	0.62	0.72	Intercept	-9.14	0.0	0.19	Temp	-0.4	0.0	0.15	RH	-0.19	0.0	0.13	Transit	0.53	0.0	0.12	Yt-1	0.56	0.0	0.08	Precision	8.82	0.0	0.28
hCoVs	AH_RH_Yt-1_Transit	-2354.0	0.63	0.89	Intercept	-9.16	0.0	0.19	AH	-0.43	0.0	0.16	RH	-0.12	0.06	0.12	Transit	0.53	0.0	0.11	Yt-1	0.59	0.0	0.07	Precision	8.86	0.0	0.28
hMPV	Yt-1	-2475.0	0.46	0.11	Intercept	-9.35	0.0	0.23	Yt-1	0.64	0.0	0.08	Precision	8.61	0.0	0.3												
hMPV	Temp_Yt-1	-2475.0	0.47	0.27	Intercept	-9.35	0.0	0.23	Temp	-0.08	0.25	0.14	Yt-1	0.65	0.0	0.08	Precision	8.62	0.0	0.3								
hMPV	AH_Yt-1	-2475.0	0.47	0.26	Intercept	-9.35	0.0	0.23	AH	-0.09	0.22	0.14	Yt-1	0.65	0.0	0.08	Precision	8.62	0.0	0.3								
hMPV	Transit_Yt-1	-2492.0	0.53	0.67	Intercept	-9.48	0.0	0.22	Transit	0.43	0.0	0.18	Yt-1	0.49	0.0	0.1	Precision	8.85	0.0	0.3								
hMPV	AH_RH_Yt-1	-2473.0	0.47	0.29	Intercept	-9.35	0.0	0.23	AH	-0.09	0.22	0.14	RH	0.06	0.45	0.15	Yt-1	0.66	0.0	0.08	Precision	8.62	0.0	0.3				
hMPV	Temp_RH_Yt-1	-2473.0	0.47	0.28	Intercept	-9.35	0.0	0.23	Temp	-0.08	0.29	0.14	RH	0.05	0.54	0.15	Yt-1	0.65	0.0	0.08	Precision	8.62	0.0	0.3				
hMPV	Temp_Yt-1_Transit	-2500.0	0.57	0.85	Intercept	-9.47	0.0	0.21	Temp	-0.22	0.0	0.13	Transit	0.49	0.0	0.17	Yt-1	0.47	0.0	0.11	Precision	8.89	0.0	0.29				
hMPV	AH_Yt-1_Transit	-2503.0	0.57	0.89	Intercept	-9.47	0.0	0.21	AH	-0.24	0.0	0.13	Transit	0.5	0.0	0.16	Yt-1	0.46	0.0	0.11	Precision	8.9	0.0	0.29				
hMPV	Temp_RH_Yt-1_Transit	-2499.0	0.57	0.82	Intercept	-9.47	0.0	0.21	Temp	-0.23	0.0	0.13	RH	-0.09	0.31	0.17	Transit	0.51	0.0	0.17	Yt-1	0.44	0.0	0.12	Precision	8.9	0.0	0.29
hMPV	AH_RH_Yt-1_Transit	-2501.0	0.58	0.87	Intercept	-9.48	0.0	0.21	AH	-0.24	0.0	0.13	RH	-0.06	0.49	0.16	Transit	0.52	0.0	0.17	Yt-1	0.45	0.0	0.12	Precision	8.91	0.0	0.29

USA (March 2020-October 2022)

Virus	Model	AIC	R^2	pValueAC	Variable1	Coef1	pValue1	error1	Variable2	Coef2	pValue2	error2	Variable3	Coef3	pValue3	error3	Variable4	Coef4	pValue4	error4	Variable5	Coef5	pValue5	error5	Variable6	Coef6	pValue6	error6
IVA	Yt-1	-2118.0	0.37	0.0	Intercept	-8.14	0.0	0.25	Yt-1	0.49	0.0	0.06	Precision	7.3	0.0	0.32												
IVA	Temp_Yt-1	-2118.0	0.38	0.0	Intercept	-8.15	0.0	0.25	Temp	-0.11	0.14	0.15	Yt-1	0.48	0.0	0.06	Precision	7.33	0.0	0.32								
IVA	AH_Yt-1	-2119.0	0.39	0.0	Intercept	-8.16	0.0	0.25	AH	-0.14	0.08	0.15	Yt-1	0.48	0.0	0.06	Precision	7.34	0.0	0.31								
IVA	Transit_Yt-1	-2117.0	0.38	0.0	Intercept	-8.14	0.0	0.25	Transit	0.05	0.44	0.14	Yt-1	0.47	0.0	0.07	Precision	7.3	0.0	0.31								
IVA	AH_RH_Yt-1	-2119.0	0.4	0.0	Intercept	-8.16	0.0	0.24	AH	-0.12	0.14	0.16	RH	-0.11	0.16	0.15	Yt-1	0.49	0.0	0.06	Precision	7.35	0.0	0.31				
IVA	Temp_RH_Yt-1	-2119.0	0.4	0.0	Intercept	-8.16	0.0	0.24	Temp	-0.12	0.13	0.16	RH	-0.13	0.08	0.15	Yt-1	0.49	0.0	0.06	Precision	7.35	0.0	0.31				
IVA	Temp_Yt-1_Transit	-2120.0	0.4	0.03	Intercept	-8.15	0.0	0.24	Temp	-0.24	0.02	0.2	Transit	0.19	0.04	0.19	Yt-1	0.42	0.0	0.08	Precision	7.35	0.0	0.31				
IVA	AH_Yt-1_Transit	-2121.0	0.41	0.03	Intercept	-8.16	0.0	0.24	AH	-0.25	0.01	0.19	Transit	0.18	0.04	0.18	Yt-1	0.42	0.0	0.08	Precision	7.36	0.0	0.31				
IVA	Temp_RH_Yt-1_Transit	-2120.0	0.41	0.04	Intercept	-8.16	0.0	0.24	Temp	-0.23	0.02	0.2	RH	-0.1	0.17	0.15	Transit	0.16	0.09	0.19	Yt-1	0.43	0.0	0.09	Precision	7.37	0.0	0.31
IVA	AH_RH_Yt-1_Transit	-2120.0	0.41	0.03	Intercept	-8.16	0.0	0.24	AH	-0.23	0.03	0.2	RH	-0.06	0.5	0.16	Transit	0.16	0.1	0.19	Yt-1	0.43	0.0	0.09	Precision	7.36	0.0	0.31
RSV	Yt-1	-1949.0	0.75	0.0	Intercept	-7.77	0.0	0.15	Yt-1	0.94	0.0	0.08	Precision	8.02	0.0	0.26												
RSV	Temp_Yt-1	-1947.0	0.75	0.0	Intercept	-7.77	0.0	0.15	Temp	0.01	0.81	0.11	Yt-1	0.93	0.0	0.08	Precision	8.02	0.0	0.26								
RSV	AH_Yt-1	-1947.0	0.75	0.0	Intercept	-7.77	0.0	0.15	AH	-0.01	0.87	0.11	Yt-1	0.94	0.0	0.08	Precision	8.02	0.0	0.26								
RSV	Transit_Yt-1	-1977.0	0.8	0.98	Intercept	-7.81	0.0	0.13	Transit	0.36	0.0	0.13	Yt-1	0.81	0.0	0.09	Precision	8.24	0.0	0.25								
RSV	AH_RH_Yt-1	-1947.0	0.75	0.0	Intercept	-7.76	0.0	0.15	AH	-0.0	0.99	0.11	RH	-0.07	0.24	0.12	Yt-1	0.95	0.0	0.08	Precision	8.02	0.0	0.26				
RSV	Temp_RH_Yt-1	-1947.0	0.75	0.0	Intercept	-7.76	0.0	0.15	Temp	0.01	0.89	0.11	RH	-0.07	0.24	0.12	Yt-1	0.95	0.0	0.08	Precision	8.02	0.0	0.26				
RSV	Temp_Yt-1_Transit	-1976.0	0.8	1.0	Intercept	-7.8	0.0	0.13	Temp	-0.05	0.33	0.1	Transit	0.36	0.0	0.12	Yt-1	0.81	0.0	0.09	Precision	8.24	0.0	0.25				
RSV	AH_Yt-1_Transit	-1976.0	0.8	1.0	Intercept	-7.8	0.0	0.13	AH	-0.04	0.36	0.1	Transit	0.35	0.0	0.12	Yt-1	0.82	0.0	0.09	Precision	8.24	0.0	0.25				
RSV	Temp_RH_Yt-1_Transit	-1974.0	0.8	1.0	Intercept	-7.81	0.0	0.14	Temp	-0.05	0.31	0.1	RH	0.03	0.59	0.12	Transit	0.37	0.0	0.13	Yt-1	0.8	0.0	0.1	Precision	8.25	0.0	0.26
RSV	AH_RH_Yt-1_Transit	-1974.0	0.8	1.0	Intercept	-7.81	0.0	0.14	AH	-0.05	0.29	0.1	RH	0.04	0.47	0.12	Transit	0.37	0.0	0.13	Yt-1	0.8	0.0	0.1	Precision	8.25	0.0	0.25
hCoVs	Yt-1	-1983.0	0.55	0.8	Intercept	-8.08	0.0	0.15	Yt-1	0.53	0.0	0.05	Precision	8.31	0.0	0.26												
hCoVs	Temp_Yt-1	-1982.0	0.56	0.72	Intercept	-8.08	0.0	0.15	Temp	-0.06	0.32	0.12	Yt-1	0.52	0.0	0.05	Precision	8.32	0.0	0.26								
hCoVs	AH_Yt-1	-1983.0	0.56	0.69	Intercept	-8.09	0.0	0.15	AH	-0.11	0.1	0.13	Yt-1	0.52	0.0	0.05	Precision	8.34	0.0	0.26								
hCoVs	Transit_Yt-1	-2015.0	0.66	0.01	Intercept	-8.15	0.0	0.13	Transit	0.26	0.0	0.08	Yt-1	0.58	0.0	0.05	Precision	8.6	0.0	0.25								
hCoVs	AH_RH_Yt-1	-2000.0	0.62	0.98	Intercept	-8.16	0.0	0.14	AH	-0.07	0.26	0.13	RH	-0.26	0.0	0.11	Yt-1	0.58	0.0	0.06	Precision	8.53	0.0	0.26				
hCoVs	Temp_RH_Yt-1	-2001.0	0.62	0.99	Intercept	-8.16	0.0	0.14	Temp	-0.09	0.14	0.12	RH	-0.27	0.0	0.11	Yt-1	0.58	0.0	0.06	Precision	8.54	0.0	0.26				
hCoVs	Temp_Yt-1_Transit	-2026.0	0.69	0.39	Intercept	-8.16	0.0	0.13	Temp	-0.22	0.0	0.12	Transit	0.3	0.0	0.08	Yt-1	0.54	0.0	0.05	Precision	8.69	0.0	0.25				
hCoVs	AH_Yt-1_Transit	-2029.0	0.69	0.49	Intercept	-8.17	0.0	0.13	AH	-0.25	0.0	0.12	Transit	0.3	0.0	0.08	Yt-1	0.54	0.0	0.05	Precision	8.72	0.0	0.25				
hCoVs	Temp_RH_Yt-1_Transit	-2039.0	0.72	0.78	Intercept	-8.2	0.0	0.12	Temp	-0.22	0.0	0.12	RH	-0.2	0.0	0.1	Transit	0.27	0.0	0.08	Yt-1	0.58	0.0	0.06	Precision	8.83	0.0	0.25
hCoVs	AH_RH_Yt-1_Transit	-2036.0	0.71	0.65	Intercept	-8.2	0.0	0.12	AH	-0.21	0.0	0.12	RH	-0.16	0.0	0.1	Transit	0.26	0.0	0.08	Yt-1	0.57	0.0	0.06	Precision	8.81	0.0	0.25
hMPV	Yt-1	-2054.0	0.51	0.17	Intercept	-8.05	0.0	0.21	Yt-1	0.63	0.0	0.07	Precision	7.54	0.0	0.29												
hMPV	Temp_Yt-1	-2052.0	0.51	0.17	Intercept	-8.05	0.0	0.21	Temp	-0.01	0.89	0.14	Yt-1	0.63	0.0	0.07	Precision	7.54	0.0	0.29								
hMPV	AH_Yt-1	-2053.0	0.51	0.16	Intercept	-8.05	0.0	0.21	AH	-0.03	0.72	0.15	Yt-1	0.63	0.0	0.07	Precision	7.54	0.0	0.29								
hMPV	Transit_Yt-1	-2097.0	0.65	0.03	Intercept	-8.21	0.0	0.18	Transit	0.38	0.0	0.1	Yt-1	0.74	0.0	0.06	Precision	7.96	0.0	0.28								
hMPV	AH_RH_Yt-1	-2052.0	0.52	0.2	Intercept	-8.06	0.0	0.21	AH	-0.01	0.92	0.15	RH	-0.1	0.19	0.15	Yt-1	0.65	0.0	0.08	Precision	7.56	0.0	0.29				
hMPV	Temp_RH_Yt-1	-2052.0	0.52	0.2	Intercept	-8.06	0.0	0.21	Temp	-0.02	0.84	0.15	RH	-0.1	0.17	0.14	Yt-1	0.65	0.0	0.08	Precision	7.56	0.0	0.29				
hMPV	Temp_Yt-1_Transit	-2104.0	0.67	0.46	Intercept	-8.24	0.0	0.18	Temp	-0.23	0.0	0.14	Transit	0.43	0.0	0.1	Yt-1	0.71	0.0	0.07	Precision	8.04	0.0	0.27				
hMPV	AH_Yt-1_Transit	-2104.0	0.67	0.43	Intercept	-8.24	0.0	0.18	AH	-0.22	0.0	0.14	Transit	0.42	0.0	0.09	Yt-1	0.72	0.0	0.06	Precision	8.04	0.0	0.27				
hMPV	Temp_RH_Yt-1_Transit	-2103.0	0.67	0.5	Intercept	-8.24	0.0	0.18	Temp	-0.22	0.0	0.15	RH	-0.04	0.58	0.13	Transit	0.42	0.0	0.1	Yt-1	0.72	0.0	0.08	Precision	8.04	0.0	0.27
hMPV	AH_RH_Yt-1_Transit	-2102.0	0.67	0.42	Intercept	-8.24	0.0	0.18	AH	-0.23	0.0	0.15	RH	0.01	0.87	0.14	Transit	0.42	0.0	0.1	Yt-1	0.71	0.0	0.08	Precision	8.04	0.0	0.27
