# Supplementary material for: The effects of weather and mobility on respiratory viruses dynamics before and during the COVID-19 pandemic in the USA and Canada
Source: PLOS Digit Health. 2023 Dec 21;2(12):e0000405. doi: 10.1371/journal.pdig.0000405 (PMC10734953; doi:10.1371/journal.pdig.0000405)
Supplement: Attachment — Submitted filename: Varela_Response_letter.pdf [file pdig.0000405.s020.pdf]

Response letter to the Editors and Reviewers

We would like to thank the editors and the reviewers for the thoughtful comments. We have prepared a revised version of the manuscript and believe to have addressed all raised concerns.

The main changes are:

- We have added three new controls for differences in the mobility data pre- and during the pandemic, which are now included in the text and in Supplementary Figures 11 and 12.
- We have repeated all the correlation analysis using Spearman correlation and added them to Supplementary Figure 7.
- We have included p-values in all the correlation analysis (main text and supplements).
- We have repeated the incidence-weather correlations including 1 and 2 weeks lag for the weather variables, and included the results in the text and in the new Supplementary Figure 6.
- We have clarified and/or expanded several sections, such as weather and mobility variable selection (Supplementary Figure 4) , exact dates for the different periods in each analysis (new Periods subsection in the Methods), choice of auto-correlation lags in the regression analysis (Supplementary FIgure 5), among others.
- We also made language improvements throughout the text.

Other points were also addressed, leading to clarifications or changes in the manuscript. These are all marked in blue. Text that remains the same but that was moved within the manuscript is marked in green.

Below, we address the points raised by the reviewers one by one. We really believe that these changes have greatly benefited the manuscript and we are grateful for the suggestions, comments, and new proposed analysis. We summarize the reviewers' main comments, offer an overall response, and then respond to each point separately.

Reviewer #1:

We would like to thank Reviewer 1 for the careful analysis, for the comments and for the recommendation to publish. Reviewer 1's main concerns regard the mobility analysis and the choice of temporal windows. We agree that both are central to our study and have revised the manuscript to clarify the methodology, added a comparison to other mobility variables and tested our models using different time windows (both in terms of time span and for the autocorrelation lags).

1.1 - While it was stated that the start date for the "pandemic period" was selected based on public declaration by the WHO, I wonder if your team considered or tested the pandemic period starting when the virus was first identified in late 2019. As this period

***from late 2019 to March 2020 did have circulation of SARS-CoV-2, I was surprised it was not included in the pandemic period*.**

This is a very interesting point as the period selection depends on whether we should expect a change in human behaviour (and, therefore, in viral transmission) being more due to the circulation of SARS-CoV2, to its prevalence, or to the awareness of that circulation. Our initial version assumed that, prior to the awareness and the first mobility limitations, we should observe none to minimal difference but, indeed, this assumption was not explicitly tested, as it should. Therefore, we repeated the analysis to include the period from January 2020 to March 2020 and a) calculated the regression coefficients for all viruses (new Supplementary Figure 11), b) found no significant difference from including this period (as the mean values calculated for one period were always within two standard errors of the mean values of the other period).

We have included the following description to the methods: "*As a control, we have tested including the period between the week of the 18th January 2020 to March 2020 in the "pandemic" period as there was already SARS-CoV2 circulation (see [65]). Furthermore, as these periods include different time-spans and dynamics, and do not represent unique epidemiological seasons, we have further repeated the analysis using similar periods of 15 months for the pandemic period, from January 2021 to March 2022*."

We have also changed the main manuscript and chose to address this point together with another relevant question from Reviewer 2, who raised concerns about the differences in period length, between the pre- and pandemic intervals.

Therefore, the main text now reads: "*As the defined pandemic period does not correspond to when the virus was first identified, but only to when the pandemic was declared (March 2020), we repeated the analysis considering that the pandemic started when SARS-CoV2 was identified and known to already be in circulation (from mid January 2020). Furthermore, and given that our pre-pandemic and pandemic periods have different durations, we also considered equivalent length-periods (15 months each) to control for possible effects of differences in period. We found no significant differences in the first case (S11 Fig and our results did not change, in the second (S12 FigA). Finally, and given the high variability present in the "number of trips" variable, during the pandemic, we also built a regression model for the whole period available, using the pandemic as a dummy variable, and found a significant interaction between the pandemic variable and the number of trips for all the virus (except IVB), supporting our previous results on the relevance of mobility for the incidence of these viruses during the pandemic (S12 FigB)*."

1.2 - Mobility data for "pre-pandemic" only includes data from late 2019 to March 2020, which, as just mentioned, is a period of circulation for SARS-CoV-2, even if the declaration had not been officially given. I found this to be a hole in the work, as mobility can not properly be addressed/assessed for pre-pandemic if this is the only window it is considered within. It is not stated specifically why mobility data was not included or considered for earlier periods.

We are unsure whether we understood this comment. In fact, we do not have good quality mobility data for Canada in the pre-pandemic period but, for the USA, the mobility data for the pre-pandemic period, spans 15 months (from January 2019 to March 2020) and that is

the period represented in Figure 5. If the comment referred to the period between January 2020 and March 2020, we believe to have addressed it in the answer to 1.1, as we now include analysis for this period for both the temperature and mobility variables.

But we agree that the mobility datasets were poorly explained (also mentioned by reviewer 3) and we have clarified the collection and selection criteria in the methods.

1.3 - Of the correlation tests completed, was there a specific reason why Pearson was selected over or not in combination with Spearman? I wonder if there was a reason to consider only linear effects and not monotonic associations.

We would like to thank the reviewer for this comment as this was something that we had indeed done and discussed internally. Our reasoning for not including it was that, as all incidences were normalized, we were more concerned with time coincidence (when peaks-co-occur) than with the rank of particular weeks. Moreover, the Spearman results were very consistent with our analysis, using only Pearson: in summary, for the pre-pandemic period, and as expected, we found that the Spearman correlations are generally stronger than the ones obtained with Pearson, have similar significances, and display the same patterns (strong correlations with weather variables temperature and AH, and two viral blocks appearing). For the pandemic period comparisons, and also similar to Pearson, we found weaker correlations between the viruses.

However, we agree that there is no strong reason for only showing Pearson correlations and we now include the Spearman analysis as Supplementary Figure 7, mention it in the methods, and added the following sentence to the main text: "*The observed correlations patterns are consistent when using Spearman correlation (S7 Fig) and do not change when considering lagged weather of up to 2 weeks (S6 Fig)*." and "*When we repeated the analysis using Spearman correlation, we found similarly weak correlations between viruses. This further supports the observation that weather played a more nuanced role on viral incidence, during the pandemic period (S7 Fig)*."

1.4 - Also for your correlations, there are many correlation tests discussed and heatmaps shown, but little if any discussion of the statistical significance of these correlations. Was there statistical significance tested for these Pearson correlation analyses?

Again, we thank the reviewer for pointing that out. We have done this analysis but indeed neglected to include it as most of the correlations are significant. We have now updated the Methods, the figure legends, Figures 2 and 4 and Supplementary Figures 2, 3, 4, 6 and 7. Significance is now identified in all figures as white (in dark background) or black (in light background) if p-value $\leqslant$0.05 and in light gray, if non-significant.

1.5 - When selecting for autocorrelation (AC) windows, I am curious of why your team only selected 1 week or 2 weeks or no autocorrelation. Did you run any tests for 3 weeks or monthly/4-week patterns? I would suggest adding information for these periods or clarifying why they are not considered.

Indeed this was not clear in the methods or the text. The lag choice was done using two rationales: what we know in terms of the dynamics of the viruses (from infection to symptoms) and what we expected in terms of reporting delays. However, it is possible that delays could be higher or that auto-correlations could have other dynamics. Therefore, we have repeated the analysis now including 3 week and 4 week lags and found that, for all tested circumstances, the best results were obtained for a lag of 1 week (higher ΔAIC). For this reason, we kept the 1-week lag in the main manuscript, mention this analysis in the methods and now present these comparisons in a new Supplementary Figure 5.

1.6 - The selection of only one primary mobility variable was not well described. Why was only "number of trips" selected for analysis? This selection of just one factor should be described more thoroughly prior to labeling as "the mobility variable". Weather factors are heavily discussed and supported, while mobility factors seemed brushed off and not fully supported or explained. Originally, upon introductory description, it seemed like mobility data and its multiple factors would play a larger role in analysis but then it did not.

We agree with the reviewer that not enough attention was given to explaining the mobility variables. We have now included further justification for the variables' choice. Therefore, the methods section now reads: "*As these include a number of metrics, Principal Components Analysis was used to select relevant mobility variables, using as criteria low correlation with other variables, and how much variability it explained, i.e., how representative of overall mobility each metric is. The variables ``Number of residents staying at home" (i.e., persons who make no trips of more than one mile away from home) and `Number of trips made by residents" (i.e., movements that include a stay of longer than 10 minutes at an anonymized location away from home) fulfilled both criteria and were selected for further analysis (S4 Fig)" and we have also added the corresponding figure (Supplementary Figure 4)*.

1.7 - In the conclusion of your regressions section, directly prior to results, you stated that there was previous evidence of the relevance of the RH AC Temperature Model. Please cite this prior work here rather than just saying there is evidence.

Indeed, this reference was missing, thank you for noticing. It was now included as: "F*or the weather-only analysis, the temperature-RH-AC model was chosen to compare the results because, first, there is previous evidence of the relevance of a combination of these two factors on the epidemiological dynamics of some respiratory virus ([16, 18]), and, second, the analysis results showed that this model represented an adequate model for comparison of all viruses, regions and periods (see Results).*"

16. Marr LC, Tang JW, Van Mullekom J, Lakdawala SS. Mechanistic insights into the effect of humidity on airborne influenza virus survival, transmission and incidence. Journal of the Royal Society Interface. 2019;16(150):20180298.

18. Lowen AC, Mubareka S, Steel J, Palese P. Influenza virus transmission is dependent on relative humidity and temperature. PLoS pathogens.2007;3(10):e151.

1.8 - In the results section, add references to the sentence "as others before us, we found strong correlations between the viruses….".

Indeed, these references were missing, thank you for noticing. They were now included after the sentence as: "*As others before us ([23, 24], we found strong correlations between the viruses, temperature and AH, but weaker or no correlation with RH.*"

23. Roussel M, Pontier D, Cohen JM, Lina B, Fouchet D. Quantifying the role of weather on seasonal influenza. BMC Public Health. 2016;16(1):1–14.

24. Deyle ER, Maher MC, Hernandez RD, Basu S, Sugihara G. Global environmental drivers of influenza. Proceedings of the National Academy of Sciences. 2016;113(46):13081–13086.

1.9 - Results for mobility being such a considerable factor for the COVID-19 pandemic period made the fact that we had no true pre-pandemic mobility data glaring and seems like a major missing key part for this analysis. Though the weather-related factors are well supported, mobility comparisons do not.

We believe to have addressed this point when answering 1.2 (as that is true for Canada but not for the USA) and apologize if this is not the case.

1.10 - Was there considerations of other NPI factors that could've been added to this analysis? The discussion of these factors helped the narrative, but I would have hoped to see at least a statement planning this integration and consideration in future work. The variability in mandates and restrictions, as mentioned, play a huge role in transmission and are a needed component for this analysis to be fully robust.

We agree with the reviewer and this is definitely a limitation of our study, together with the fact that we are doing national instead of regional analysis.

We have expanded this discussion, and the current version now reads: "*We note that this study was done at the country level and that mobility is likely to play an even more visible role when analyzing outbreaks at higher geographical granularity [91], as in USA and, to some extent, in Canada, mobility restrictions were implemented and adjusted at the state or provincial levels. Similarly, several other NPIs (including masking policies) often followed different strategies, with each territory having their own guidelines and regulations. Ideally, future studies should take regional differences into consideration and recent data sets [92] might allow a more detailed analysis of the role of different NPIs. Still, it should be noted that, even where similar NPIs were in place, it is not trivial to quantify population adherence or disentangle their effects. Overall, mobility data has been shown to be a good proxy for the strength of lockdown measures and other NPIs [49, 50] and has been increasingly available and used in recent years as an indicator of behavioural changes affecting infection dynamics (e.g. [51–54]).*"

1.11 - While the relevance for climate change and for broad understanding of disease transmission represent important precipitators to this type of analysis, it would be helpful

to see additional support for the "so what" of this factor analysis. Really hammer home why this work is important – because it is!

We appreciate the comment and fully agree that a lot still needs to be done to understand the intricate relationship between transmission, human behaviour, and climate change. What we see is that temperature correlates very well with transmission pre-pandemic, but that correlation disappears during the pandemic; and instead human motility seems to be playing a larger role. So there is an interaction between temperature and behaviour, which is now made clearer in Supplementary Figure 12.

In order to predict the effects of climate change on transmission, we would need to make guesses as to how humans will behave. Will they tend to be out more in winter because it is warmer? Or will they stay home more often during extreme weather?

The best we can do with our current knowledge and results is simulate multiple scenarios and analyze the consequences. We believe that is a very timely analysis but beyond the scope of the current paper but now also suggest that further studies are required and the discussion includes the following paragraph: "F*urthermore, as summer peaks are clearly possible for some of them, global changes in weather might alter viral dynamics in ways that are difficult to predict, which is particularly relevant in the context of climate change [79, 80]. Further analysis on the effects of weather and behaviour on NIRVs is in order, both in laboratory and epidemiological settings, to inform models and allow simulation of different complex scenarios and interactions.*"

1.12 Again, while these are changes I feel would really help the flow and impact of the work, I overall feel the analyses were sound and that the narrative is well-written and impactful.

We again thank the reviewer and hope to have addressed all main concerns.

Reviewer #2:

We would like to thank Reviewer 2 for the comments and for the suggestions. Reviewer 2's main concerns regard the temporal analysis (lags and duration of the analyzed periods) and we have significantly revised the manuscript according to the raised issues.

2.1 The paper reads overall well. However, it is too lengthy and, in many parts, redundant. The description of the methods is in part repeated on the Results. In addition, the results are sometimes discussed and interpreted in the Results section. I believe that the paper would be much more readable if the Results section was more succinct with no repetition of methods and no anticipation of the discussion.

We apologize for the lack of clarity and find that this is a common difficulty, as often the results from one method/analysis inform the choice of the following methods and analytical tools and, conversely, some detail of the methodology is important to help interpret specific

results. We have tried to improve both sections for brevity and clarity and all introduced changes are marked in blue.

2.2 The study is conducted at the country level. Both the USA and Canada are extended countries. In the USA, weather variables vary substantially across different regions. Authors should properly acknowledge this limitation and discuss how this impacts the results.

We agree with the reviewer that this is an important point and a limitation of our study, together with the within-country variability in the implementation of mobility restrictions and other NPIs (see reply to reviewer 1 point 10).

We have included this in the discussion that now reads: "*The effect of weather in the pre-pandemic period is significant even though this study was conducted at a national level and there is great variability in temperature, humidity and other weather variables within countries, especially in the case of the USA. Further studies could benefit from the regional level data available to investigate the specific effects of different climate types on the incidence of these viruses.*"

2.3 Do the Authors consider a lag in the correlation analysis? I believe that given the delays in reporting the correlation analysis could consider a lag of at 1 or 2 weeks.

This is a very relevant point and we thank the reviewer for it. As we had included lags in the regression analysis and a one-week lag systematically offered a better AIC (please also see our answer to Reviewer 1, point 5), we have repeated the correlations including 1 and 2 weeks lag. These results are now shown in the Supplementary Figure 6 and mentioned in the methods and results sections. We found no significant differences between the different lags (after correcting for multiple hypothesis testing), most likely because both weather and incidence have high auto-correlation.

2.4 Although mobility data for the USA are available only for one year during the pre-pandemic period, it seems to me that the range of variation of mobility during the pandemic period is greater than in the pre-pandemic period. This could explain why this quantity was more important in explaining the incidence trend during COVID-19 pandemic. The Authors should discuss this point more in detail.

This is a crucial issue that we had neglected to control for, and would like to thank the reviewer for pointing it out. We have interpreted the comment in two different ways as this difference in periods (pre- and pandemic) is relevant on both dimensions: looking at, for example, Supplementary Figure 2, both the lengths (x-axis, as reviewer 1 also pointed out), and the variation (y-axis) are different, with a larger amplitude in the pandemic mobility data.

Therefore, we have addressed both.

First, we repeated the analysis using equal periods (15 months each, please also see answer to R1, point 1) and found no significant difference when shortening the pandemic period to

January 2021 to March 2022, whether analyzing the "number of trips" or the "population at home" variables. We have added the corresponding analysis to the supplements (Supplementary Figure 12 A and B) and discuss it in the manuscript. Second, and using normalized incidence, weather and mobility data as before, we created a dummy variable for the pandemic period and repeated the analysis as a single regression spanning the whole time period. As can be seen in Supplementary Figure 12 C, there is a significant interaction between the pandemic variable and the number of trips, indicating that indeed the effect of the number of trips on incidence changes with the pandemic. Therefore, the manuscript now reads in the methods section: *"As a control, we have tested including the period between the week of the 18th January 2020 to March 2020 in the "pandemic" period as there was already SARS-CoV2 circulation (see [65]). Furthermore, as these periods include different time-spans and dynamics, and do not represent unique epidemiological seasons, we have further repeated the analysis using similar periods of 15 months for the pandemic period, from January 2021 to March 2022. 189 Finally, for the mobility analysis with the COVID-19 Community Mobility Reports the pandemic period starts on the week of the 14th March, 2020, and ends in March 2022."* And in the results section: *"As the defined pandemic period does not correspond to when the virus was first identified, but only to when the pandemic was declared (March 2020), we repeated the analysis considering that the pandemic started when SARS-CoV2 was identified and known to already be in circulation (from mid January 2020). Furthermore, and given that our pre-pandemic and pandemic periods have different durations, we also considered equivalent length-periods (15 months each) to control for possible effects of differences in period. We found no significant differences in the first case (S11 Fig) and our results did not change, in the second (S12 FigA). Finally, and given the high variability present in the ``number of trips" variable, during the pandemic, we also built a regression model for the whole period available, using the pandemic as a dummy variable, and found a significant interaction between the pandemic variable and the number of trips for all the virus (except IVB), supporting our previous results on the relevance of mobility for the incidence of these viruses during the pandemic (S12 FigB)."*

2.5 "it was suggested that the IVB/Yamagata lineage could have become partially extinct, due to the lack of animal reservoirs and other epidemiological characteristics" I find this sentence quite vague. I don't believe that the lack of animal reservoirs is the main cause of IVB/Yamagata extinction. In addition, "other epidemiological characteristics" is not clear.

We agree that this sentence does not add much and is too vague and has been removed from the manuscript.

2.6 "Furthermore, if some of these viruses are less weather-sensitive than previously believed, and summer peaks are clearly possible for some of them, global changes in weather might alter viral dynamics in ways that are difficult to predict, which is particularly relevant in the context of climate change": Authors should better elaborate on this point since it is quite important, but at the same time, this sentence does not seem to me well substantiated.

Indeed this was not properly phrased, as reviewer 1 also pointed out, and was answered above (point 1.11).

Again, we thank Reviewer 2 for the comments and hope to have addressed all concerns.

Reviewer #3:

We would like to thank Reviewer 3 for the comments, which mostly focus on lack of clarity or poor writing, from our end. Although some of the simplicity was deliberate, as we do not wish to overclaim our results or hint at certainty where there is little, we definitely agree with all the reviewers that the writing could be refined and have made several changes to the manuscript to improve language and clarity.

3.1. The study title should clearly define that this study includes data from the US and Canada.

We agree that the title was too vague and it now reads: "The effects of weather and mobility on respiratory viruses dynamics before and during the COVID-19 pandemic in the USA and Canada."

3.2. The abstract should be more informative and scientifically based. The current version seems to be too simple and plain.

Unfortunately, it was not very clear to us exactly how the abstract should be improved, but we have made some changes aiming at better clarifying the claims.

3.3. It will be better if the text is more professional. So please consider changes e.g., in line 35 where the word "Naturally" seems to be inappropriate.

We thank the reviewer for the comment and have made several changes to the manuscript, to remove this and other adverbs of manner (striked-out).

3.4. Data source and management is a little bit unclear. How do the different data collection and reporting methods affect the results? What is the risk of bias and how do the Authors handle it?

We would like to thank the reviewer for this comment as the same issue was raised by reviewer 1 and agree that this was not clear in the methods.

We now include a paragraph that states: "*The main criteria for data collection was availability. We 1) selected USA and Canada as these countries collect respiratory viral data throughout the year (and not only during ``influenza-season") and continued to do so using similar surveillance systems during the COVID-19 pandemic, minimizing measurement bias; 2) included all respiratory viruses that were collected by both countries, for the maximum period of time available, and 3) included pandemic mobility data for both USA and Canada and also pre-pandemic mobility in the case of the USA (unavailable for Canada).*"

We also expanded the discussion on using national vs. state/province data and on the lack of pandemic mobility data for Canada.

3.5. Why only temperature and humidity were selected? The methods section should be more clear and easy to follow. Some assumptions may be unclear to the readers. How about the meteorological data for Canada?

The Iowa Environmental Mesonet shares weather information from several stations around the world, including the US and Canada. So, as stated in the methods, we have the same variables for both countries.

We agree that different measurements, such as wind or rain, may be relevant for transmission. We now clarify our choice of humidity and temperature. In particular, the methods section now reads:

We now add: "*The weather variables Temperature (T) and Relative Humidity (RH) were selected for 1) being previously shown to affect virus transmission and survival [21, 23, 24, 61] and 2) being easy to obtain from different locations in a standardized way*." and "*Absolute humidity (AH, gr/m3), which has also been shown to affect virus transmission and survival [15, 23, 24],was estimated following Eq (2)*"

3.6. The Authors used two different approaches and data collection methods for two different countries. There is a serious risk of bias. The Authors may focus only on 1 country and should consider a separate analysis for the US or Canada.

We thank the reviewer for this comment as this is a central issue that we clearly failed to convey. We would like to clarify that, as discussed in point 4, we use the exact same methodology for both countries throughout the paper. The only relevant difference is the lack of mobility data for Canada in the pre-pandemic period. Therefore, the mobility comparative analysis (pre- and pandemic) is only possible for the USA. But, using the same Google mobility dataset, we show that, during the COVID19 pandemic, the weight of mobility in viral dynamics was similar in the USA and in Canada (Figure 6): the variable visitors to transit stations had higher regression coefficients than the variable temperature, for all viruses analyzed.

We hope to have clarified this in the answer to point 4 but will be happy to further improve this, if necessary.

3.7. Wording like "it is quite possible" or "is reason to believe that" seems inappropriate in the scientific paper.

Again, we thank the reviewer for the careful reading and have minimized such wording.
